# Men talk less than women during multitasking

**DOI:** 10.1007/s00426-026-02279-5

**Published:** 2026-05-15

**Authors:** André J. Szameitat, Diana P. Szameitat

**Affiliations:** 1 Department of Psychology, Centre for Cognitive and Clinical Neuroscience, Brunel University of London, Uxbridge, UK; 2 Department of Psychology and Neuroscience, City St George’s, University of London, London, UK

**Keywords:** Sex differences, Gender differences, Multitasking, Dual-task performance, Conversation behaviour, Talkativeness, Stereotype

## Abstract

Contrary to the widespread stereotype that women are better at multitasking than men, no consistent sex differences in multitasking abilities between males and females have been found. We developed a novel multitasking paradigm consisting of five tasks designed to simulate real-life scenarios. In Study 1, 41 males and 37 females (the ‘multitaskers’) performed similarly in all tasks except a conversation task, which males ignored more than twice as often as females. To test whether this sex difference is noticeable to others, in Study 2 160 naïve observers watched videos of the multitaskers and rated their appearance. Females were rated as being more in control of the task, performing better, and being less stressed than males. Importantly, these ratings were strongly influenced by the conversation task performance. Therefore, this study not only showed a sex difference in multitasking performance but also that this difference impacts how multitaskers are perceived by others, providing a potential explanation for the development of a stereotype that women are better at multitasking than men.

**Supplementary Information:**

The online version contains supplementary material available at 10.1007/s00426-026-02279-5.

## Introduction

Multitasking, which is performing two or more tasks at the same time or frequently switching between tasks, has become a ubiquitous characteristic of modern life. For example, multitasking is needed for driving (Engström et al., 2013; Nijboer et al., 2016), in many occupations (Chesley, 2014; Franksiska & Yuniawan, 2023; Paridon & Kaufmann, 2010), and when dealing with household and childcare (Szameitat et al., 2015). Many people engage in multitasking even in their leisure time (Ophir et al., 2009; Sanbonmatsu et al., 2013; Strobach et al., 2012). There is a widespread stereotype that women are better at multitasking than men (Strobach & Woszidlo, 2015; Szameitat et al., 2015). However, contrary to such a stereotype, past research only found small and inconsistent sex differences in multitasking abilities (Buser and Peter 2012; Colom et al. 2010; Himi et al. 2023; Hirnstein et al. 2019; Hirsch et al. 2019; Laguë-Beauvais et al. 2013; Lui et al. 2021; Mantyla 2013; Redick et al. 2016a; Ren et al. 2009; Stoet et al. 2013; Strayer et al. 2013), raising the question whether there are notable sex differences in multitasking abilities at all. While stereotypes can develop without underlying differences (Allport, 1954), the fact that *“stereotype accuracy is one of the largest and most replicable findings in social psychology”* (Jussim et al., 2015, p. 490) favours an alternative explanation, namely that sex differences in multitasking do exist, but that they have not yet been identified. The goal of the present study was to shed new light on sex differences in multitasking and on possible explanations why a potential stereotype might have developed.

Real-life multitasking is often highly stressful, as most parents can attest when they coordinate household chores such as cooking and looking after one or more toddlers at the same time (Szameitat et al., 2015). Everyday multitasking often requires interleaving and switching between tasks (e.g., reading a recipe, handing a toddler a toy, resume reading the recipe) as well as performing tasks concurrently (e.g., monitoring a toddler and talking to a family member while chopping vegetables) (Burgess et al., 2000; Hirnstein et al., 2019). Such everyday multitasking situations contrast sharply with previous research on sex differences in multitasking, where participants were exclusively seated at a computer and engaged in various cognitive tasks (see Himi et al., 2023 for a review). To enhance ecological validity and better reflect real-life multitasking, the present study examined sex differences using a complex multitasking paradigm that more closely replicated the demands of everyday multitasking than any previous research (cf. Himi et al., 2023).

While potential sex differences in stressful real-life multitasking can be expected in any task, a more specific suggestion can be found in the stereotype mentioned in the popular book “Men are from Mars, Women are from Venus” (Gray, 2002), which states the following behaviour of males under stress: *“When a man is stressed he will withdraw into the cave of his mind […] At such times*,* he becomes increasingly distant*,* forgetful*,* unresponsive*,* and preoccupied in his relationships. For example*,* when having a conversation with him at home*,* it seems as if only 5% of his mind is available for the relationship […]”* (Gray, 2002, p. 33), while the behaviour of females is described as “*[…]*,* talking is a natural Venusian [female] reaction to stress.*” (Gray, 2002, pp. 37–38; cf. also Taylor et al., 2000). To our knowledge, the only available evidence (tentatively) supporting these statements comes from a study by Stoet et al. (2013), who employed a simple conversation task in which participants were required to provide single-word responses to general knowledge questions during a concurrent simulated telephone call. Their findings suggested that indeed females might be better at multitasking when a conversation task is involved (numerically, with p-values approaching significance, e.g. *p* = .06). It is conceivable that in a more demanding multitasking situation, in particular including a more complex conversation task, potential sex differences could be more pronounced. Therefore, we hypothesized that during demanding everyday multitasking, men might, among other differences, specifically reduce the amount of conversation they engage in, i.e. they might talk less. To test for this, our multitasking paradigm included a rather complex conversation component. If men do indeed reduce their talkativeness while multitasking, it could negatively impact others’ perceptions of their overall multitasking abilities. More specifically, a poorer performance on the conversation task might be noticeable to observers, and based on the horn effect (i.e., reverse-halo effect, Sattler et al., 1970; Thorndike, 1920), these observers could form negative impressions of men’s multitasking abilities as a whole. To test whether performance in any of the tasks constituting the multitasking paradigm influences how observers view the multitaskers, we videorecorded the participants while multitasking and, in Study 2, presented the recordings to naïve observers and asked them to rate the multitaskers according to stress level, estimated performance, and the level of control they have over the task, among further items.

For the present study, we developed a paradigm that mimics real-life multitasking more closely than previous studies in that area. In more detail, participants had to work on five non-computerised tasks spread out across three different tables. The main task was a simulated Cooking Task in which participants had to follow a recipe using pretend cooking utensils and ingredients (primary task; performed at one table). Participants were instructed to interrupt the cooking tasks when a kitchen timer rang and to work briefly on two paper-and-pencil tasks (Phone-Number Search Task and Visual Search Task, mimicking cognitively demanding interruptions to a primary task; both at a second table) before being allowed to return to the cooking task. Throughout these three tasks, participants had to concurrently monitor a slideshow presenting words on different coloured backgrounds on a screen for occasional targets (a word presented on red background) and write them down at a third table (Monitoring Task, mimicking e.g. watching that a toddler does not do mischief) and also had to verbally answer complex pre-recorded questions played via speakers, such as “Would you rather lose all of your money and valuables or all of the pictures you have ever taken, and why?” (Conversation Task, one question every 20 s). In everyday life, the time pressure under which multitasking is performed can vary between a relaxed slower pace and a stressful higher pace, depending on the exact situational context. To simulate this difference, the time pressure was gradually increased throughout the 10-min lasting trial by setting the timer to successively shorter intervals, which allowed to test whether sex differences depend on the pace and stress-level of the multitask (see Methods for details).

## Study 1

The aim of Study 1 was to test whether sex differences exist in multitasks mimicking real-life scenarios more closely, with a particular focus on the conversation task.

### Methods study 1

#### Participants

In Study 1, we planned to test 50 males and 50 females (sex defined as biological sex at birth) for 80% power to detect a medium effect (d = 0.5, α = 0.05) using independent-samples t-tests. Due to the Covid pandemic, fewer participants were tested (41 males, 41 females), and some data were lost. The final analysis included 37 females (M = 23.62 years, SD = 6.51) and 41 males (M = 23.13 years, SD = 2.88).For one of the key findings (sex difference in the Conversation Task) we observed an effect size of Cohen’s d = 0.611, and a post-hoc power-analysis with the actual participant numbers revealed an achieved power of 0.76 (alpha = 0.05; two-sided).

Participants were recruited locally from Brunel University of London campus and received in total £36 for participation (£10/hour; the data presented here are part of a larger study). Both studies were approved by the Department of Life Sciences ethics committee, Brunel University London, UK, and data were collected in accordance with the declaration of Helsinki. All participants gave written informed consent before participation and were debriefed in the end. In Study 1, participants were informed about the video recordings in the Participant Information Sheet and consented explicitly to the point of “I agree to be video recorded while doing some of the experimental tasks and I agree that excerpts of these recordings may be presented to participants in future studies.”

#### Materials and tasks

**Fig. 1:**
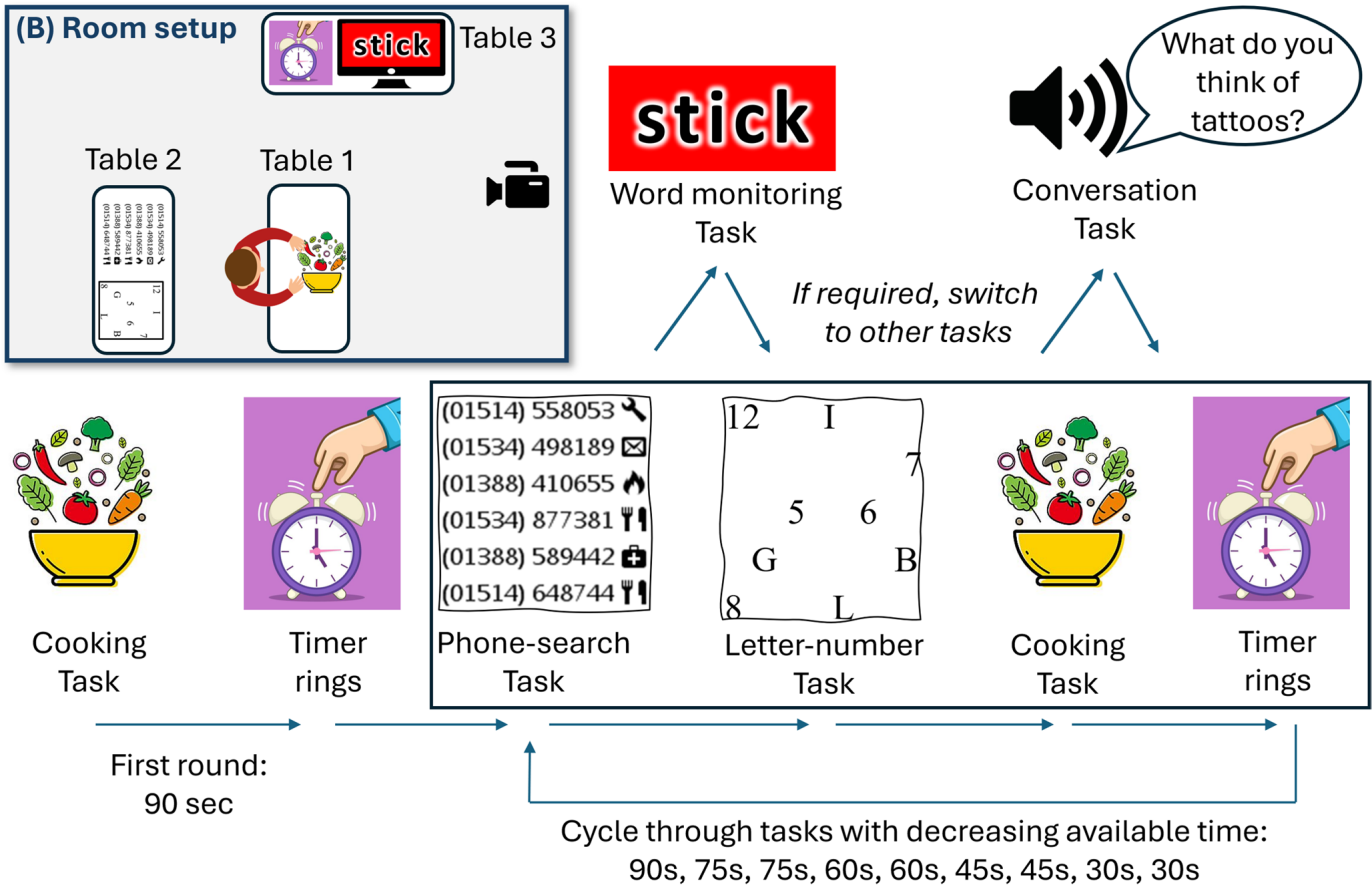
Experimental design. (B) Location of the tables and camera in the testing room

The multitask consisted of five different tasks (Fig. 1).

##### **Task 1**,** Cooking task**

The cooking task was described as the main task to participants. They had to follow a printed recipe and were given a plastic bowl and a variety of pretend ingredients, such as wooden and glass beads of different colours and little cards depicting e.g. 100 g of flour, red pepper, and 10 g of butter, which had to be added to the bowl. The wooden beads had to be beaded onto a string for some steps of the recipe. This task was performed on table 1. Participants were informed that they would be scored based on how far they progressed in the recipe and how many mistakes they made.

##### **Task 2**,** Phone number search task**

Participants were given a folder with a couple of sheets filled with phone numbers which consisted of an area code, the actual number, and a little icon depicting the nature of the business (e.g. knife and fork for restaurants). Participants had to search through the sheet for a specific combination, e.g. restaurants with an area code of 01421. If they found a number, they had to write it down on paper and continue to the next task (the number-letter task, see below). For good performance, participants had to keep in mind which part of the sheet they already searched through and from where to pick up the next time (participants were not able to write on the sheet with the phone numbers). The phone search task and the number-letter task were both done on table 2, located behind table 1.

##### **Task 3**,** Number-letter task**

Participants were given a pen and a sheet with 12 letters (A – L) and 12 numbers (1–12) randomly distributed across the sheet (similar to the Trail Making Test Part B). On each round, they had to tick off a set of letters and numbers, i.e. A and 1 in the first round and B and 2 in the second round, and so forth. Again, for good performance it was useful to remember where they had left off in the previous round.

##### **Task 4**,** Word monitoring task**

A computer screen was placed well in sight of the participants on table 3 to the front/side of table 1. A slideshow was run which showed a new slide every 25 s. Each slide showed one word in an easy-to-read big font on different coloured backgrounds. Participants had to write down only those words which were presented on a red background. There were 24 slides in total, 9 with a red background, i.e. on average a target appeared every 67s. They had to monitor the screen throughout all the above tasks and, if necessary, interrupt them, walk to table 3 where the screen was placed, write down the word on paper, and then resume the task they had to interrupt.

##### **Task 5**,** Conversation task**

Pre-recorded brief questions were presented every 20 s via speakers (first question after 20s into the trial). Most questions were phrased in a way to invite longer answers, e.g. “Would you rather always be 10 minutes late or always be 20 minutes early, and why?”. Participants were asked to answer the questions properly as if they were part of a conversation, i.e. they should not give simple one-word answers.

#### Procedure

Participants were instructed and practiced all tasks. Participants were informed that the tasks would be scheduled by a kitchen timer. They would start with the cooking task until the kitchen timer would ring. Then they had to reset and restart the kitchen timer (by pressing the ‘Start’ button on the timer twice), go to the second table, find the next number in the number-search task, and then find the next set of the number-letter task. Only then they were allowed to return to the cooking task and spend the remaining time (before the next ringing of the timer) on the cooking task. During all these tasks, they had to monitor the screen for a word on red background (and, if necessary, interrupt the current task to write down the word at table 3), and to verbally answer the questions of the conversation task. There were no chairs, participants were standing for all tasks, and they had to walk between the three tables.

In the first two rounds, the timer rang every 90 s. In the next two rounds, this was reduced by the experimenter to increase time pressure, and the timer rang after 75 s, then two rounds of 60 s, two rounds of 45 s, and finally two rounds of 30 s each, resulting in an overall trial duration of 10 minutes. We used two timers in the laboratory room and every time the time limit was reduced, the experimenter prepared the currently non-used timer to the new time limit. They pressed the Start button on the timer when the participant pressed the Start button on their timer, and then the experimenter swapped the timer. This procedure was demonstrated to participants before the experiment started. Because the experimenter managed the timers, the participants always only had to press the Start button twice, but never to set times themselves. For the shorter durations, some participants sometimes had only a couple of seconds left on the Cooking Task (the instructed main task) before the timer rang again.

Throughout the session and after the session, the experimenters made notes and comments of the participant’s performance. In particular, the experimenters manually recorded how many questions in the conversation task were answered. Each session was conducted by one of seven different experimenters.

#### Video recordings

Participants were video-recorded during the whole task in 4k resolution using a Panasonic HC-VX870 camcorder.

See Supplementary Information for more methodological details of Study 1.

### Results study 1

Thirty-seven females (mean age 23.62 years, SD 6.51) and 41 males (mean age 23.13 years, SD 2.88) were analysed. To allow for direct comparisons between tasks despite different scales of the tasks, scores were normalised (across male and female participants) separately for each task, so that positive scores reflect a better performance than the overall average (and negative scores worse performance than the overall average).

#### Sex differences in multitasking

**Fig. 2:**
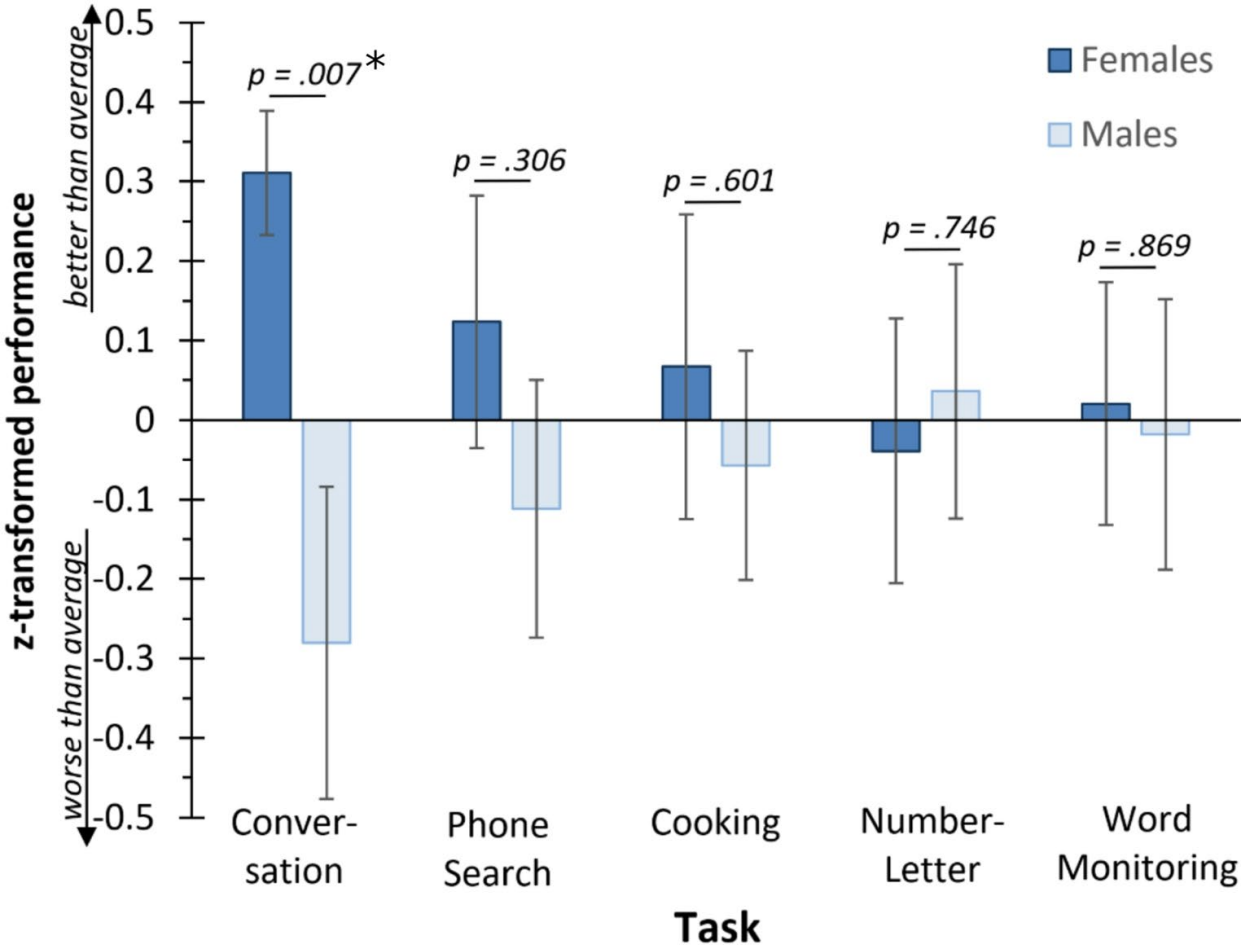
Performance of female and male participants in the five different tasks constituting the multitask. P-values indicate significant differences between males and females (independent samples t-tests). * Bonferroni-corrected *p* = .035. Error bars denote SEM according to the logic of Loftus and Masson (1994)

Because we were interested whether sex differences might show specifically in the Conversation Task, as hypothesised above, we tested for sex differences in each task separately (Fig. 2). Indeed, a significant sex difference was apparent in the Conversation Task. On average, females answered 24.76 out of the 28 questions (SD 3.63), while males answered 20.24 questions (SD 9.58), which was significantly different (independent samples t-test (unequal variances) t(52.279) = 2.80, *p* = .007, p (Bonferroni-corrected for 5 comparisons) = 0.035, Cohen’s d = 0.611; Bayes Factor BF_10_ = 5.049). In other words, females did not answer 11.6% of the questions, while males did not answer more than twice as many questions, namely 27.7%. In the remaining four tasks, no significant sex differences were observed (Phone Number Search Task: t(76) = 1.032, *p* = .306, Cohen’s d = 0.234, BF_10_ = 0.373, BF_01_ = 2.681; Cooking Task: t(72) = 0.525, *p* = .601, Cohen’s d = 0.123, BF_10_ = 0.271, BF_01_ = 3.690; Number-Letter Task: t(75) = 0.324, *p* = .746, Cohen’s d = 0.074, BF_10_ = 0.247, BF_01_ = 4.049; Word Monitoring Task: t(75) = 0.165, *p* = .869, Cohen’s d = 0.038,, BF_10_ = 0.239, BF_01_ = 4.184; see Supplementary Information).

#### Quality and speed of conversation task answers

Males might not only answer less questions in the Conversation Task, but the answers given may also be of lower quality. However, analyses (See Supplementary Information) showed that the quality (i.e., whether answers were brief one-word answers or long answers as in a conversation) and speed (i.e., the delay between end of the question and start of the answer) of the answers did not differ significantly between males and females (Quality: t(63) = 1.389, *p* = .170, Cohen’s d = 0.346, BF_10_ = 0.574, BF_01_ = 1.742; Speed: t(65) = 0.057, *p* = .955, Cohen’s d = 0.014;, BF_10_ = 0.251, BF_01_ = 3.984). Therefore, our findings suggest that during multitasking males answer significantly fewer questions in a conversation task, but *if* they answer, their answers have a comparable quality and speed compared to those of females.

#### Participants’ perception of the multitasking paradigm

We asked the multitasking participants after the task how they perceived the task and how they felt. There were no sex differences for the questions (1–9 point Likert scale) asking whether the task required multitasking (females = 8.4, SD 0.98; males = 8.0, SD 1.79; independent-samples t-test t(66) = 1.064, *p* = .291, Cohen’s d = 0.258, BF_01_ = 2.481), whether the task was difficult (females = 6.6, SD 2.13, males = 6.4, SD 2.15; t(65) = 0.486, *p* = .629, Cohen’s d = 0.119, BF_01_ = 3.610), whether they enjoyed the task (females = 7.5, SD 2.06, males = 7.6, SD 1.78; t(66) = 0.321, *p* = .749, Cohen’s d = 0.078, BF_01_ = 3.846), and whether they are now (after doing the task) tired (females = 3.9, SD 2.16, males = 4.3, SD 2.53; t(64) = 0.629, *p* = .532, Cohen’s d = 0.155, BF_01_ = 3.356) and can concentrate well (females = 5.6, SD 1.90, males = 5.6, SD 2.29; t(66) = 0.104, *p* = .918, Cohen’s d = 0.025, BF_01_ = 4). This shows that self-reported experience of the multitasking performance was comparable between males and females.

### Discussion study 1

Four of the five tasks comprising the multitask, which simulates real-life situations, showed no statistically significant sex differences. However, the Conversation Task showed a significant sex difference, with males failing to answer more than twice as many questions as females. Although males answered less questions than females, the quality and swiftness of the answers did not differ between sexes.

While there is a wealth of research into multitasking, only few studies have investigated sex differences. The vast majority of these studies used computerised cognitive paradigms, such as the task-switching paradigm (Hirsch et al., 2018; Stoet et al., 2013), the dual-task paradigm of the psychological refractory period (Hirsch et al., 2018; Laguë-Beauvais et al., 2013), a combined Flanker and Go/No Go task (Ren et al., 2009), or complex working memory span tasks (Redick et al. 2016b). Some studies used self-developed paradigms, such as Mantyla (2013).

There are a few studies which aimed to investigate specifically real-life or everyday multitasking, e.g. the Edinburgh Virtual Errand Task (Logie et al., 2011), a conference planning task (Hirnstein et al., 2019), and tasks aiming to mimic the demands of pilots (Santiago-Espada et al., n.d.), for a review see (Himi et al., 2023). However, even those multitasks labelled as everyday-life-mimicking tasks were done in front of a computer with a single screen. They were usually called everyday-life tasks because they were more complex, up to a level which is somewhat similar to gaming, such as the conference planning task where an avatar has to be moved around to prepare a room for a conference meeting.

Finally, there is the domain of research into driving, in particular investigating the multitasking situation of driving while holding a conversation (either to a passenger or via cell phone). While this research is also a good simulation of real-life multitasking, unfortunately this literature is not informative for the present purposes, because most studies did not test for sex differences (e.g., Beede & Kass, 2006; Boboc et al., 2022; Burns et al., 2002). The few studies which did test for sex differences did so only for the driving task, but not for the conversation ‘task’ (e.g., Cao & Liu, 2013; Collet et al., 2010; Drews et al., 2008). One reason for this is that the demand of talking to someone else (passenger/phone) was often not operationalised as a task and, thus, it was not assessed with respect to performance, such as the quality of the conversation.Taken together, the vast majority of prior studies are basic cognitive paradigms based on speeded choice-response tasks and even prior everyday-multitasking paradigms remain computerized tasks, albeit of greater complexity. The only area which indeed mimics everyday life multitasking closely (talking while driving) is not informative for sex differences in multitasking because they were focussing only on one task of the multitasking situation, i.e. driving, and not all tasks constituting the multitasking situation.

While we are not aware of a prior study which would have used a comparable conversation task, there are studies which used auditory and/or verbal versions of cognitive tasks, such as the operation span task (Lui et al., 2021). Sometimes, only task instructions were given verbally (Hirnstein et al., 2019), and sometimes participants had to speak out the task instructions and answers (Lui et al., 2021). In these tasks, no sex differences were observed. We believe that the reason for this is that those tasks were basically standard cognitive tasks (e.g. memory tasks) with verbal answers but not complex verbal tasks in which statements about one’s own opinions had to be expressed in one or more sentences (Charlton, 2009; Collet et al., 2010; Drews et al., 2008). We believe that our Conversation Task lies somewhere between more cognitive verbal tasks and natural longer conversations with several turns of the conversation partners. Future studies need to investigate whether the currently observed sex difference in the Conversation Task is the same, amplified, or attenuated in a true real-life conversation.

The reasons why men did not answer the Conversation Task more often than women is unclear. One possibility is that they missed the question in the first place, e.g. because they were too focussed on the other tasks. An alternative explanation is that they made a conscious choice of not answering the question. Maybe they deemed the Conversation Task as less important and wanted to maintain better performance in the other tasks and, for that, were willing to sacrifice performance in the Conversation Task. However, given that performance in the other tasks was comparable (and numerically women were better), this would mean that men needed more attentional resources than women for comparable performance in the other tasks.

After the time limit, which gradually decreased from 90 s to 30 s in steps of 15 s, the participants had to reset and restart the timer by pressing the timer’s start button twice. We decided to operationalise this additional demand not as a separate task. One reason was that we did not have meaningful data for this task, because the timer was loud and always acted upon by participants, i.e. performance was 100% accurate. Another way to analyse performance related to the timer could be to measure the time it took participants to stop the timer after it started ringing. However, first, this was not measured and, second, this time was influenced by various factors, such as where exactly in the laboratory room the participant was located when the timer started to ring. Nevertheless, the resetting of the timer could be considered a sixth task in our multitasking paradigm.

Taken together, Study 1 found that in an everyday-life mimicking multitasking scenario, women performed significantly better in the Conversation Task than men. Such performance differences can potentially explain the development of a stereotype that women are better at multitasking than men. However, it is also possible that observers base their judgement about multitasking abilities of a person not on the multitasker’s task performance, but on other aspects of their appearance, e.g. how stressed they appear. For example, there are sex differences in stress levels when mathematics tasks are performed, while there are no sex differences in the actual math performance (Devine et al., 2012). Similarly, males or females may be perceived to be calmer or more stressed during multitasking, although many performance measures do not differ between sexes. Therefore, the aim of Study 2 was to test how naïve observers rate male and female multitaskers when they watch them multitasking and whether actual task performance of the multitaskers affects the observer ratings.

## Study 2

### Introduction study 2

While we observed a sex difference in the Conversation Task performance in Study 1, it is an open question whether this performance difference actually influences how observers perceive the multitaskers and, thus, whether it contributes to a stereotype that women are better at multitasking at all. Therefore, Study 2 aimed at investigating whether observers perceive multitasking females and multitasking males differently. To test for this, the participants of Study 1 (from now on called the ‘multitaskers’) were recorded (video and audio) while performing the five tasks of the multitasking paradigm. Participants of Study 2 (the ‘observers’, naïve to the research question) watched excerpts of these recordings and were asked to evaluate the multitasker in the video according to several criteria, such as how stressed the person in the video appeared, or whether they appeared to be in control of the task or not. We expected that the ratings would reflect that overall women are perceived to be better at multitasking than men. In addition, because the performance in the Conversation Task was very prominent and noticeable to observers, we expected that better Conversation Task performance would be associated with more positive ratings, such as being less stressed and more in control of the task.

### Methods study 2

#### Participants

In Study 2, 80 males (mean age 34.15 years, SD 5.74) and 80 females (mean age 35 years, SD 6.86) were recruited as observers via Testable Minds (https://minds.testable.org/) and tested online using Psytoolkit (Stoet, 2010, 2017). The study took approx. 50 min and was reimbursed with US$8.

#### Materials

The 10-minute multitasking trial of each multitasker in Study 1 was audio- and videorecorded (see Study 1 for details). For Study 2, two 75 s long clips were extracted from each 10-minute recording from each multitasker. The first clip started 55 s after the start of the 10-minute trial, and the second clip showed the last 75 s of the trial. Because time pressure for the multitaskers increased during the trial (by means of shorter intervals of the timer, see Study 1 for details), we labelled the first clip Low Time Pressure (LTP) and the second clip High Time Pressure (HTP). Each clip showed the multitaskers performing all task and also included how the multitaskers were walking between tables (the tasks were spread across different tables, see Study 1 for details).

Due to the turmoil left by the sudden onset of the Covid pandemic (e.g., no backup of some recording sessions, some student research assistants leaving the country before handing back paper forms), the available data of video recordings does not fully overlap with the behavioural data of Study 1. We had one LTP and one HTP video clip of 80 multitaskers, resulting in a stimulus set of 160 video clips.

#### Procedure

The task of the participants (the ‘observers’) in Study 2 was to watch the 75-second video clips of the multitaskers from Study 1 (presentation of videos was pseudo-randomised, see Supplementary Materials for details). For time reasons, the whole stimulus set of 160 videos was split and presented in eight runs of the experiment with 20 videos each, using PsyToolkit (Stoet, 2010, 2017). There were 20 observers (10 male) in each run, i.e. each of the 160 video clips was rated by 20 observers. Each observer watched and rated 20 video clips out of the stimulus set of 160 videos. These 20 clips came from five male and five female multitaskers (with the LTP and HTP clips of each multitasker being included). The experiment lasted approx. 50 min.

After viewing each video clip, observers responded to seven rating questions using a continuous slider scale ranging from 0 to 100. Observers were asked to “Use the slider to indicate how the person in the video appeared to you”, followed by the seven sliders of (1) Calm [0] to Stressed [100], (2) Struggled with task [0] to Was well in control of task [100], (3) Disliked doing the task [0] to Liked doing the task [100], (4) Performed poorly [0] to Performed well [100], (5) Put no effort into the task [0] to Put a lot of effort into the task [100], (6) Sleepy/Tired [0] to Wide-Awake/Alert [100], and (7) Sad [0] to Happy [100].

After providing informed consent, observers were instructed and practiced the rating task with two videos which were not part of the main experiment. To avoid triggering of existing potential stereotypes, observers were not informed about the research question, and the term ‘multitasking’ or related terms were not used. Observers were told the main task was a ‘Mixing Task’ instead of a Cooking Task to avoid priming of sex stereotypes (see Supplementary Materials for full information on the instructions).

After all videos had been rated, observers were asked some further questions. In more detail, we asked whether the participants believed men or women were better at multitasking by asking “What do you think, are men or women better at multitasking? (select “50” if you think both are the same)” (scale 0–100). Note that this question was presented after observers rated the videos, i.e. towards the end of the experiment. Therefore, this question cannot have affected the ratings. Furthermore, we asked “When you evaluated the people in the videos, what did you base your evaluation/rating on?” with the answer options Yes/No and the items (multiple answers possible) “The expression in their face”, “The way they moved their arms”, “The way they moved their whole body (while standing)”, “The way they walked from one task to the next”, “What they were saying and how they were saying it”, plus an open text field.

See Supplementary Information for more methodological details of Study 2 (including a photo of the experimental setup and the full instructions given to observers).

#### Statistics

To investigate the effects of multitaskers’ Sex and Time Pressure on observer ratings, we calculated repeated-measures 2 × 2 ANOVAs with the factor Sex (male vs. female multitasker) and the factor Time Pressure (Low Time Pressure, LTP vs. High Time Pressure, HTP). To analyse whether the observer ratings were associated with the performance in the Conversation Task, we calculated Pearson’s correlations between rating scores and Conversation Task performance.

### Results study 2

#### Video ratings

After viewing each video, observers were asked to rate (scale 0–100) how the person in the video appeared to them in regards to: (1) Calm/Stressed, (2) Struggled with task/Was well in control of task, (3) Disliked doing the task/Liked doing the task, (4) Performed poorly/Performed well, (5) Put no effort into the task/Put a lot of effort into the task, (6) Sleepy, Tired/Wide-Awake, Alert, and (7) Sad/Happy.

**Fig. 3:**
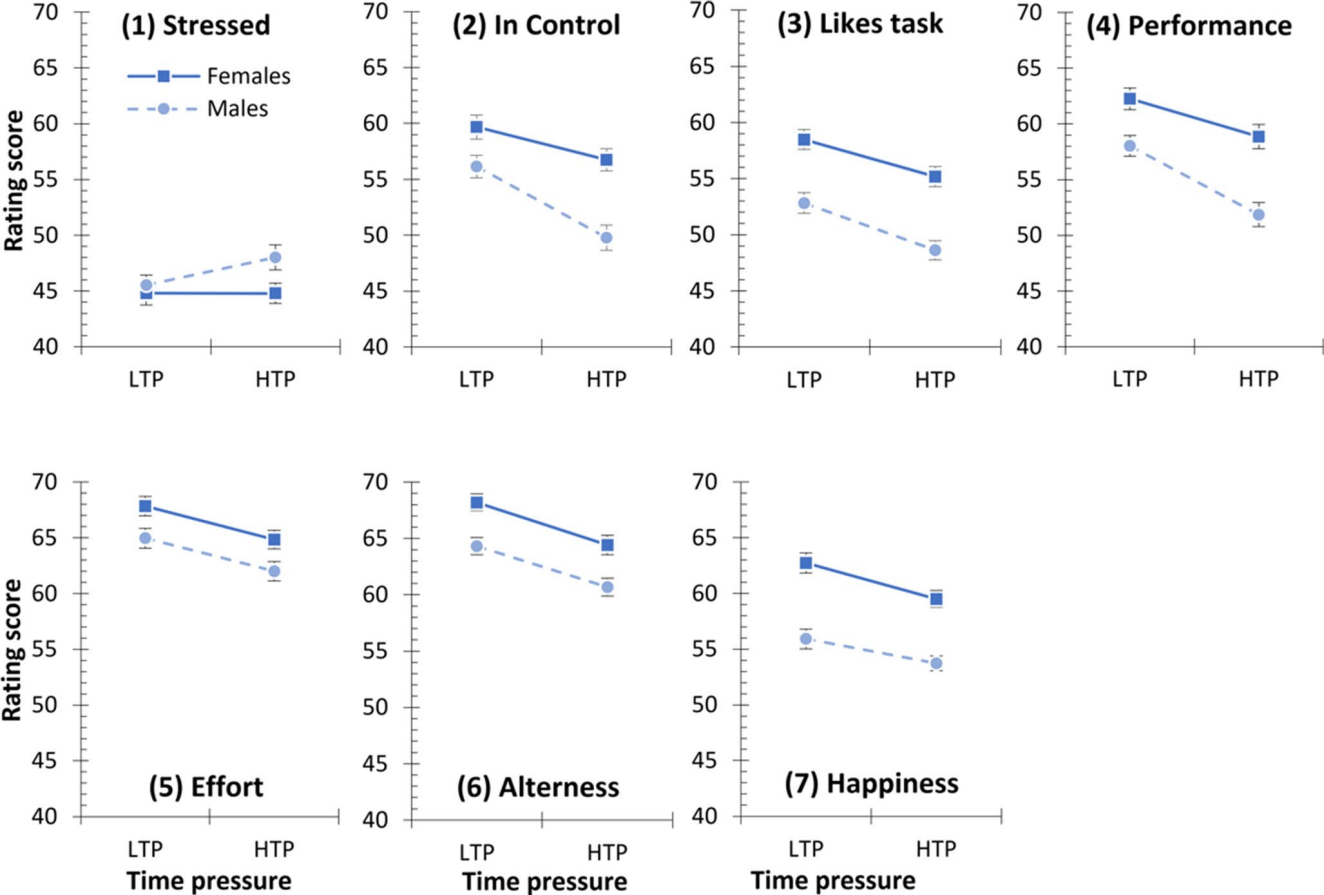
Observer ratings of the female (solid line) and male (dashed line) multitaskers performing under low time pressure (LTP) and high time pressure (HTP). Error bars denote SEM according to the logic of Loftus and Masson (Loftus & Masson, 1994)

First, we tested whether the sex of the multitasker and the time pressure affected how observers rated the multitaskers. To test for this, for each of the seven rating questions, we calculated a 2 × 2-factorial ANOVA with the factors Sex (female vs. male multitasker) and Time Pressure (LTP vs. HTP), see Fig. 3. For clarity and ease of interpretation, the results of these seven ANOVAs for the rating questions are presented together below, organized according to the two main effects and their interaction.

The main effect of Sex was significant in six out of the seven ANOVAs. In more detail, as compared to male multitaskers, female multitaskers were rated as (2) more in control of the task, (3) liking the task more, (4) performing better, (5) putting more effort into the task, (6) being more alert, and (7) being happier (all F(1, 159) > 15.458, all *p* < .001, all pη^2^ > 0.089; for full details see Supplementary Information). Only for question (1), how calm or stressed the multitasker appears, the main effect of Sex failed to reach significance (F(1,159) = 3.675, *p*=.057, pη^2^ = 0.023).

The main effect of Time Pressure was significant in six out of the seven ANOVAs. In more detail, as compared to low time pressure (LTP), under high time pressure (HTP) multitaskers were rated as (2) less in control of the task, (3) liking the task less, (4) performing worse, (5) putting less effort into the task, (6) being less alert, and (7) being unhappier (all (F(1, 159) > 25.988, all *p* < .001, all pη^2^ > 0.140; for full details see Supplementary Information). Again, for question (1), how calm or stressed the multitasker appears, the main effect of Time Pressure failed to reach significance (F(1,159) = 3.774, *p*=.054, pη^2^ = 0.023).

The interaction between Sex and Time Pressure was significant in three out of the seven ANOVAs. For question (1) how calm/stressed the multitasker appeared, high time pressure (HTP) made only males appear more stressed as compared to low time pressure (LTP), but not females, who appeared as calm in low as in high time pressure (F(1,159) = 3.939, *p*=.049, pη^2^ = 0.024). For question (2) how much in control of the task the multitasker appears, the decrease in rated control in HTP as compared to LTP was significantly stronger for males than for females (F(1,159) = 9.928, *p*=.002, pη^2^ = 0.059). Similarly, for question (4) how well the multitasker performed in the task, the decrease in rated performance in HTP as compared to LTP was significantly stronger for males than for females (F(1,159) = 6.181, *p*=.014, pη^2^ = 0.037). All other interactions were not significant (all F(1, 159) < 1.595, all *p*>.209, all pη^2^ < 0.010). Taken together, the significant interactions show that the sex differences in the ratings are more pronounced under high than under low time pressure.

Overall, these findings support our hypothesis that in our multitasking paradigm women are rated to be better at multitasking than men. This effect is often even more pronounced if the time pressure is increased.

#### Conversation task performance and ratings

The above ANOVAs tested whether the observer ratings differed between male and female multitaskers and between low and high time pressure. A further aim of this study was to test whether the observer ratings would be associated with the multitaskers’ actual performance in the Conversation Task. To test for this, we correlated multitaskers’ performance in the Conversation Task in Study 1 with the scores of the seven rating questions made by the observers in Study 2 (Table 1, see Supplementary Figure S8 for scatterplots). Pearson’s correlations showed that with better Conversation Task performance, multitaskers were rated as being more in control of the task (Pearson’s *r* = .559 ; *p* < .001; R^2^ = 0.312), as showing higher performance (*r* = .541 ; *p* < .001; R^2^ = 0.292), as being less stressed (*r* = − .450; *p* < .001; R^2^ = 0.202), as liking the task more (*r* = .561; *p* < .001; R^2^ = 0.315), and as being happier (*r* = .459, *p* < .001), while it had no significant effect on the rating of the amount of effort (*r* = .132 ; *p* = .284; R^2^ = 0.017) and the alertness (*r* = .185, *p* = .130). When the observer ratings of Study 2 were correlated with the performance of the other tasks from the multitasking paradigm in Study 1 (Cooking Task, Phone-Number Search Task, Number-Letter Task, and Word Monitoring Task), no significant correlations were observed (all *r* < .160, all *p* > .197; Table 1). Taken together, the Conversation Task performance seems to have influenced how observers perceived the multitaskers.

**Table 1:** Correlations of the performance in the five tasks of the multitasking scenario in Study 1 with the observer ratings in Study 2. Across all participants, slightly different N are caused by missing performance data in Study 1

	Conversation *N* = 68 ^(*)^	Cooking *N* = 65	Phone Number *N* = 68	Number-Letter *N* = 67	Word Monitoring *N* = 68
Question 1 Stress	*r*=-.450 *p*<.001	*r*=.037 *p*=.772	*r*=-.120 *p*=.331	*r*=-.002 *p*=.989	*r*=.057 *p*=.645
Question 2 In Control	*r*=.559 *p*<.001	*r*=.142 *p*=.256	*r*=.079 *p*=.520	*r*=-.019 *p*=.613	*r*=-.063 *p*=.613
Question 3 Liking	*r*=.561 *p*<.001	*r*=-.059 *p*=.641	*r*=.037 *p*=.762	*r*=-.112 *p*=.366	*r*=.005 *p*=.966
Question 4 Performance	*r*=.541 *p*<.001	*r*=.068 *p*=.591	*r*=.101 *p*=.410	*r*=-.074 *p*=.554	*r*=.036 *p*=.771
Question 5 Effort	*r*=.132 *p*=.284	*r*=.066 *p*=.600	*r*=-.034 *p*=.782	*r*=-.160 *p*=.197	*r*=.041 *p*=.744
Question 6 Alertness	*r*=.185 *p*=.130	*r*=.024 *p*=.847	*r*=-.071 *p*=.563	*r*=-.156 *p*=.208	*r*=.025 *p*=.844
Question 7 Happy	*r*=.459 *p*<.001	*r*=.010 *p*=.937	*r*=-.025 *p*=.840	*r*=-.123 *p*=.322	*r*=-.002 *p*=.987

(*) For the correlation between the Conversation Task performance and the questions, three datapoints were identified as outliers (z-scores of 3.59, 3.59 and 4.11) and removed from the analyses. However, results do not change if the outliers are not removed.

Note: The performance data in the tasks are taken from Study 1 and reflect performance across the whole 10-minute lasting trial. The ratings of the questions from Study 2 referred to two video clips of 75s each (LTP and HTP). For this correlational analysis, we first averaged the ratings for the LTP and HTP clip of each multitasker for one average score per multitasker

#### Observer stereotypes and ratings

It is conceivable that some of the observers themselves believed in stereotypes about sex differences in multitasking abilities, and that these stereotypes may have affected their ratings. To rule this out, the above described seven 2 × 2 mixed ANOVAs with the factors Sex and Time Pressure were conducted without observers that believed in sex differences in multitasking. In more detail, we included only those observers who answered the question “What do you think, are men or women better at multitasking? (select “50” if you think both are the same)” with a score between 49 and 51 (scale 0–100). This left 52 of the original 160 observers. Despite a much smaller sample size (*N* = 52 vs. *N* = 160), the overall pattern of results remained the same (see Supplementary Materials for full statistics), showing that the sex differences in the ratings cannot be explained by stereotypes of the observers.

#### Observers’ strategies in rating the videos

In an exploratory analysis, we assessed which aspects of the videos the observers used to rate the videos. The most used aspect, used by 86.25% of the observers, was the way multitaskers were walking from one task to the next. The expression in the multitaskers’ faces and of what they were saying and how they were saying it were each used by 75% of observers. 71.25% of observers used the movement of the whole body while the multitasker was standing and, finally, 42.5% said they had used the way they moved their arms. These data suggest that observers tried to form a rather holistic impression of the multitaskers to rate them.

#### Consistency of judgements

To test whether the different observers agreed in their judgements, we calculated the Intraclass Correlation Coefficient (ICC; absolute agreement, average measures; based on the 20 ratings given by each observer) for question 1 (calm/stressed) and question 2 (In control), separate for each of the eight runs of the experiment. For question 1, the average ICC was 0.817 (ranging between 0.676 and 0.880 across the eight runs), for question 2 it was 0.820 (ranging between 0.676 and 0.865). In particular, when considering that each experimental run had a different participant sample and used different videoclips, consistency can be considered to be very good.

### Discussion study 2

In Study 2, naïve observers watched video clips of the multitasking participants of Study 1. Observers rated females to be more in control of the task, like the task more, perform better, put more effort into the task, more alert and happier. When time pressure increased, males appeared more stressed, while females were rated as calm as under low time pressure. Furthermore, while both males and females were rated to show less control of and less performance in the task under High as compared to Low Time Pressure (HTP vs. LTP), this decrease was significantly stronger in males.

While Study 1 showed that males performed significantly worse in the Conversation Task than females, it could not answer the question whether this difference in performance is associated with how observers evaluate multitaskers. Study 2 showed that the observer ratings highly correlated with the multitaskers’ Conversation Task performance. This strongly suggests that observers did indeed notice the lower performance of the males in the Conversation Task and that it influenced their ratings across a variety of aspects (being in control of the task, task performance, stress level, liking of the task, invested effort), as would be expected from the presence of a horn effect (reverse halo effect). Therefore, the sex differences in conversation behaviour during multitasking had a profound impact on how observers viewed multitaskers in general and constitute a potential reason why a stereotype that women are better at multitasking than men might have developed.

When the observers were asked what they based their ratings on, the most common aspect was how participants walked between the different tables, followed by what and how they spoke, and the facial expression. This is noteworthy, because it is a possible explanation why previous studies have not observed the current effects. In more detail, prior studies, which were all computer-based or based on paper-and-pencil tasks (performed on a single table) had the participants just seated and did not use a conversation task. The current data do not allow to distinguish whether the walking aspect was relevant only for the observers to form their impressions, or whether it also impacted the multitaskers, e.g. by inducing the additional demand of full-body motor control and navigation between tables.

## General discussion

This study used a complex multitasking paradigm simulating common everyday multitasking. When multitaskers had to coordinate five different tasks, male multitaskers ignored the Conversation Task more than twice as often as females, while performing comparably to females in all other tasks. When naïve observers watched these multitaskers, they rated male multitaskers as being less in control of the task, performing worse, using less effort, being less alert, less happy and as liking the task less, as compared to female multitaskers. Under high as compared to low time pressure, males were perceived to experience a greater loss of control and performance, as well as a higher increase in stress than females. Consequently, sex differences were more pronounced under high time pressure than under low time pressure. These effects were strongly associated with the performance in the Conversation Task.

We propose that specifically the Conversation Task showed sex differences because of its complex conversational nature, i.e. listening and processing questions about one’s opinions and providing justified verbose answers (cf. Collet et al., 2010). The only study investigating a remotely similar paradigm was done by Stoet et al. (2013) who used a simple conversation task in which participants had to provide single-word answers to knowledge questions during a simulated phone call. Their task may lie between our complex Conversation Task and previous studies with non-conversational auditory tasks (Lui et al. 2021; Redick et al. 2016a), which generally did not report sex differences. Stoet et al. (2013) found indications of females being better at multitasking (numerically, with p-values approaching significance, e.g. *p* = .06), suggesting that the likelihood to observe sex differences may increase with the complexity of the conversation task. Since our Conversation Task still lacked important components of everyday conversations, such as managing turn-taking (Collet et al., 2010; Drews et al., 2008), we propose that sex differences in conversation behaviour during multitasking may be even more pronounced in real life than reported by us.

A reason why females were better in the conversation task could be that females have been found to be more talkative than males, in particular in self-disclosure activities (many of our questions asked about personal views, so they may fall into this category) (Leaper & Ayres, 2007; Tidwell et al., 2025). This aligns with evolutionary theories such as the Hunter-Gatherer Hypothesis, the Social Bonding Theory, and the Tend-and-Befriend Theory, which all predict that females engage more in social conversation behaviour than males (Dunbar, 1996; Geary, 1998; Silverman & Eals, 1992; Taylor et al., 2000). Therefore, females might be better specifically at conversation tasks during multitasking due to an evolutionary advantage.

In everyday life, sex difference in conversation behaviour during multitasking may show as females talking to people while multitasking, while males may reduce their talkativeness. Study 2 has shown that this does not go unnoticed, and the reduced talkativeness likely caused males to be rated as being worse at multitasking in several aspects, such as being less in control of the multitask, more stressed, and showing worse performance. Therefore, males ignoring conversations during multitasking more than females constitutes a valid potential reason for the development and maintenance of a stereotype that women are better at multitasking than men.

Reduced verbal communication among males during complex multitasking might have important workplace implications, especially in roles that depend on effective verbal interaction. While standardized procedures, such as those between pilots and control towers, are well-trained, reduced speech may be problematic in novel or critical situations. Future research should examine whether this effect of reduced talkativeness persists when conversation is more important and whether training helps maintaining verbal abilities. Another implication for the workplace is that reduced communication may be perceived as impolite or even rude unless the reason for the reduced communication is understood (Drews et al., 2008), and our findings suggest that heightened multitasking demands may be such a reason.

The four tasks in which we did not observe significant sex differences (Phone Number Search Task, Number-Letter Task, Word Monitoring Task, Cooking Task) are visual tasks with manual responses and, thus, might be more comparable to prior cognitive research using task-switching and dual-tasking paradigms, which also reported absent or rather small sex differences (Hirsch et al., 2019; Laguë-Beauvais et al., 2013; Mantyla, 2013; Ren et al., 2009; Stoet et al., 2013). This aligns with research on perceptual-motor speed and executive functions coordinating two or more visual-manual tasks, which also found only small and inconsistent sex differences (Gaillard et al., 2021; Halpern, 2013; Szameitat et al., 2002).

Taken together, our data confirm that there are no substantial sex differences in cognitive visual-manual tasks, but that significant sex differences do exist in the ability to hold a conversation while multitasking. This is an ability highly salient in everyday life and, thus, could explain the development of the widespread public stereotype that women are better at multitasking than men.

## Supplementary Information

Below is the link to the electronic supplementary material.

Supplementary Material 1

## Data Availability

The data that support the findings of this study are openly available in Figshare at 10.17633/rd.brunel.31834639.
