# Supplementary material for: Men talk less than women during multitasking
Source: Psychol Res. 2026 May 15;90(3):93. doi: 10.1007/s00426-026-02279-5 (PMC13179209; doi:10.1007/s00426-026-02279-5)
Supplement: Supplementary file 1 — Supplementary Material 1 [file 426_2026_2279_MOESM1_ESM.docx]

Supplementary Online Materials for

**Men talk less than women during multitasking**

Contents

[1. Additional Methods Study 1 1](#_Toc200011878)

[2. Additional Results Study 1 2](#_Toc200011879)

[2.1 Other parameters of the Conversation Task performance 2](#_Toc200011880)

[2.2 Participants’ perception of the multitasking paradigm 3](#_Toc200011881)

[2.3 Correlations among tasks 3](#_Toc200011882)

[2.4 Raw performance data in the five different tasks 4](#_Toc200011883)

[3. Additional Methods Study 2 5](#_Toc200011884)

[3.1 Procedure 5](#_Toc200011885)

[3.1.1 Instructions 5](#_Toc200011886)

[3.1.2 Practice 8](#_Toc200011887)

[3.1.3 Balancing 8](#_Toc200011888)

[4. Additional Results Study 2 9](#_Toc200011889)

[4.1 Full Statistics and graphs for the observer-rating analyses 9](#_Toc200011890)

[4.1.1 Question 1 (Calm vs Stressed) 11](#_Toc200011891)

[4.1.2 Question 2 (In Control): 12](#_Toc200011892)

[4.1.3 Question 3 (Likes task): 13](#_Toc200011893)

[4.1.4 Question 4 (Performance): 14](#_Toc200011894)

[4.1.5 Question 5 (Effort): 15](#_Toc200011895)

[4.1.6 Question 6 (Alertness): 16](#_Toc200011896)

[4.1.7 Question 7 (Happiness): 17](#_Toc200011897)

[4.2 Correlations between Conversation Task performance and ratings 17](#_Toc200011898)

# Additional Methods Study 1

The current study was part of a larger study, in which participants visited the lab on three different occasions (session duration 1h – 1.5h) to engage in various other tasks and questionnaires (which is why they were paid £32 in total, they received the equivalent of £10/hour). These tasks were either fully computerised or traditional paper-and-pencil tasks (e.g. Stroop task) performed sitting at a desk and have not been published yet. The everyday multitask was independent from the other tasks, no other real-live simulating task was conducted, and no cross-task influences were expected.

# Additional Results Study 1

## Other parameters of the Conversation Task performance

Our main analyses are based on the number of questions in the Conversation Task which had not been answered at all by the participants. However, it is conceivable that there are also sex differences in the questions which had been answered. To test for this, the same rater as above used the video-recordings (the 75s LTP and the 75s HTP clips) to judge the quality (i.e., whether answers were brief one-word answers or long answers as in a conversation) and speed (i.e., the delay between end of the question and start of the answer) of the answers. Quality of answers did not differ significantly between females (2.87, SD .73; on a Likert scale from 1 (very poor) to 5 (very good)) and males (2.60, SD .85; independent samples t-test, t(63) = 1.389, t-test p = .170, Cohen’s d = .346, BF_10_ = .574, BF_01_ = 1.742). Similarly, the speed by which the answers were given (on a Likert scale from 1 (slow) to 3 (fast)) did not differ significantly between females (2.26, SD .44) and males (2.25, SD .58; independent samples t-test, t(65) = .057, p = .955, Cohen’s d = .014; , BF_10_ = .251, BF_01_ = 3.984). Therefore, our findings suggest that during multitasking males answer significantly fewer questions in a conversation task, but *if* they answer, their answers have a comparable quality and speed compared to those of females.

## Participants’ perception of the multitasking paradigm

We asked the multitasking participants after the task how they perceived the task and how they felt. There were no sex differences for the questions (1 – 9 point Likert scale) asking whether the task required multitasking (females = 8.4, SD .98; males = 8.0, SD 1.79; independent-samples t-test t(66) = 1.064, p = .291, Cohen’s d = .258, BF_10_ = .403, BF_01_ = 2.481), whether the task was difficult (females = 6.6, SD 2.13, males = 6.4, SD 2.15; t(65) = .486, p = .629, Cohen’s d = .119, BF_10_ = .277, BF_01_ = 3.610), whether they enjoyed the task (females = 7.5, SD 2.06, males = 7.6, SD 1.78; t(66) = .321, p = .749, Cohen’s d = .078, BF_10_ = .260, BF_01_ = .260, BF10 = 3.846), and whether they are now (after doing the task) tired (females = 3.9, SD 2.16, males = 4.3, SD 2.53; t(64) = .629, p = .532, Cohen’s d = .155, BF_10_ = .298, BF_01_ = 3.356) and can concentrate well (females = 5.6, SD 1.90, males = 5.6, SD 2.29; t(66) = .104, p = .918, Cohen’s d = .025, BF_10_ = .250, BF_01_ = 4). This shows that self-reported experience of the multitasking performance was comparable between males and females.

## Correlations among tasks

Performance in the five different tasks, calculated across all participants, did not correlate with each other, except that the Number-Letter and the Phone-Number Search Task correlated with each other (r -.359, p = .003; Table S1). This suggests that the five different tasks overall demanded rather different, non-overlapping cognitive functions. The only exception are the Number Letter Task and the Phone Search Task, but a correlation seems reasonable, given that both are rather similar paper-and-pencil visual search tasks, which were also both performed at the same table.

**Table S1.** Cross-correlation matrix of the performance in the five different tasks

|  | Cooking | Phone-Number | Number-Letter | Word-Monit. | Conversat. |
| --- | --- | --- | --- | --- | --- |
| Cooking |  | r=-.022  p=.862  N=65 | r=.024  p=.848  N=65 | r=-.099  p=.435  N=64 | r=.043  p=.734  N=65 |
| Phone | r=-.022  p=.862  N=65 |  | r=.359  p=.003  N=67 | r=.016  p=.901  N=67 | r=-.100  p=.419  N=68 |
| Number-Letter | r=.024  p=.848  N=65 | r=.359  p=.003  N=67 |  | r=.147  p=.240  N=66 | r=.062  p=.617  N=67 |
| Word Monitoring | r=-.099  p=.435  N=64 | r=.016  p=.901  N=67 | r=.147  p=.240  N=66 |  | r=.053  p=.671  N=67 |
| Conversation | r=.043  p=.734  N=65 | r=-.100  p=.419  N=68 | r=.062  p=.617  N=67 | r=.053  p=.671  N=67 |  |

## Raw performance data in the five different tasks

To streamline the analyses, we calculated a single measure for each task, based on the correct performance minus potential errors. All data in the main manuscript and the reminder of the Supplementary Materials are based on these single measures. For the Cooking Task, this was the number of steps they managed in the recipe minus the errors they made (e.g. using a wrong ingredient or skipping a step). For the Phone Number Search Task, this was the number of correctly identified numbers, minus incorrectly identified numbers (false positives). For the Number-Letter Task, these were the correctly crossed out items minus skipped items. For the Word Monitoring Task, these were the correctly identified words on a red background minus incorrectly identified words (non-red background, i.e. false positives). For the Conversation Task, this was the number of answered questions (out of 28). The following table shows the raw data for these tasks, illustrating that the same pattern of results can be observed in the raw data as well:

| **Task** | **DV** | **Females** | **Males** | **t-test** | **p** |
| --- | --- | --- | --- | --- | --- |
| *Cooking* | *N* | *34* | *41* |  |  |
|  | # steps | 32.8 (15.1) | 31.3 (12.5) | t(73) = .454 | .651 |
|  | # Errors | 3.0 (3.9) | 3.5 (5.2) | t(73) = .469 | .640 |
|  | Steps – Err | 29.7 (15.0) | 28.1 (12.2) | t(73) = .525 | .601 |
|  |  |  |  |  |  |
| *Phone Search* | *N* | *37* | *41* |  |  |
|  | # numbers | 7.32 (2.71) | 6.85 (2.32) | t(76) = .827 | .411 |
|  | # Errors | 2.59 (3.59) | 3.10 (3.81) | t(76) = .598 | .551 |
|  | Numb – Err | 4.72 (4.00) | 3.76 (4.30) | t(76) = 1.03 | .306 |
|  |  |  |  |  |  |
| *Number-Lett.* | *N* | *37* | *41* |  |  |
|  | # ticked off | 13.1 (5.2) | 13.5 (5.4) | t(76) = .399 | .691 |
|  | # Errors | .35 (.86) | .37 (.73) | t(76) = .081 | .936 |
|  | Ticks – Err | 12.7 (5.4) | 13.1 (5.4) | t(76) = .352 | .746 |
|  |  |  |  |  |  |
| *Word monitor.* | *N* | *36* | *41* |  |  |
|  | # words | 4.75 (1.99) | 4.76 (2.23) | t(75) = .012 | .990 |
|  | # Errors | .11 (.40) | .19 (.40) | t(75) = .920 | .361 |
|  | Words – Err | 4.64 (1.87) | 4.56 (2.23) | t(75) = .165 | .869 |
|  |  |  |  |  |  |
| *Conversation* | *N* | *37* | *41* |  |  |
|  | # answered | 24.76 (3.63) | 20.24 (9.58) | t(52.3) = 2.80^(*)^ | **.007** |

(*) Levene’s test indicated unequal variances, so that corrected values have been used. If equal variances are assumed, t(76) = .269, p = .009.

Note: t-tests refer to independent-samples t-tests comparing females vs males. # is number (count).

# Additional Discussion Study 1

Multitasking performance is typically evaluated in terms of so-called multitasking costs, which refer to the performance decrements observed when a task is executed as part of a multitasking scenario, compared to when it is performed in isolation as a single task (Szameitat et al., 2011). Due to time constraints, we were not able to test all five tasks of the multitask individually as single tasks. However, this should not have affected the interpretation of the sex difference in the Conversation Task, because the task on its own would have been extremely easy. 🡨 ??? what is that? Don’t understand the logic. In the Conversation Task, one question was asked every 20 seconds. When performed on its own without any distractions, there is no reason to assume that participants would miss a question, i.e. performance can be assumed to be perfect for all participants. In such a situation, the raw performance in the multitasking scenario is equivalent to multitasking costs. 🡨 all das raus

# Additional Methods Study 2

## Procedure

### Instructions

Participants (‘observers’) first were told they will have to watch videos and that they will have to answer seven questions after each video. Then they received information about what the multitaskers in the videos had to do. In these instructions, we aimed to avoid the term multitasking (or related concepts) and other terms potentially priming sex stereotypes (e.g., we called the “Cooking Task” the “Mixing Task” to avoid observers thinking females might be better at it).


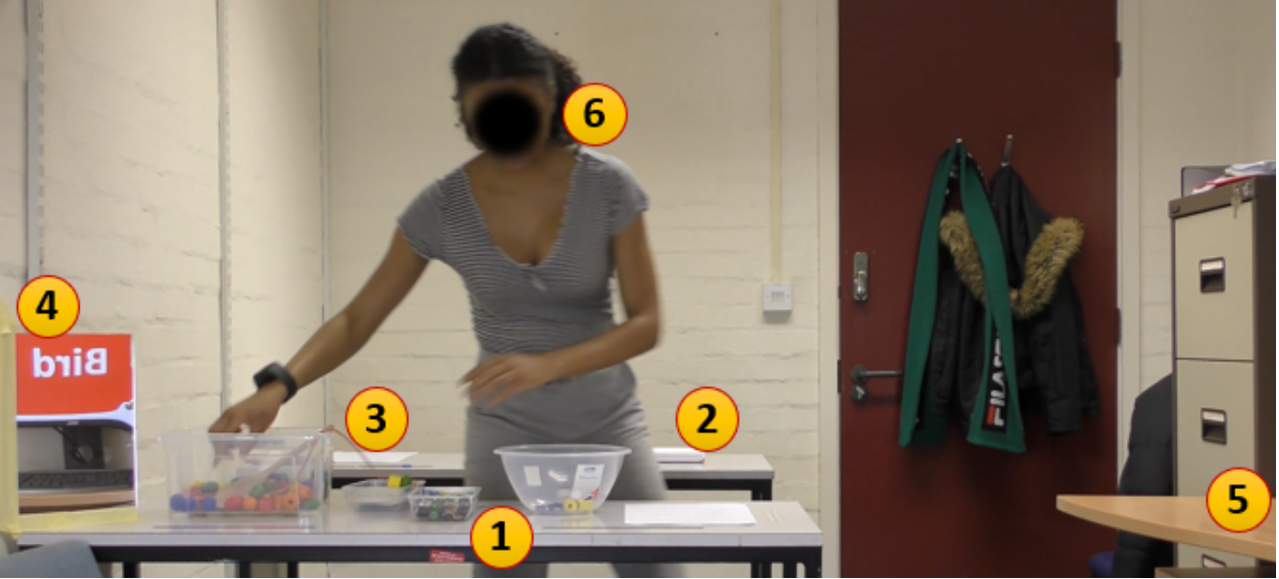


**Figure S9.** Image used for instructing observers what to do. The multitasker’s faces were visible to participants, only anonympised for publication (multitaskers consented to being video recorded and their recordings used for future experiments, see Study 1 Methods).

Literal instructions given to the participants (in the experiment this was presented directly below Figure S9):

*In the videos (the above picture shows a scene from a video), people will do a set of different tasks:

- (1) Their main task is a "Mixing" task, where they have to follow a prescribed sequence of putting several things into a bowl, threading correctly coloured beads on a string, and alike. (#1 in the picture)

- (2) When a timer beeps, they have to re-set the timer and then to move to the next task (#2, at the back table), which is finding a telephone number from a phone book and writing it down

- (3) After this, they have to do a memory task (#3 in the picture). When they are done with this, they can return to the Mixing task (#1)

- (4) They also have to monitor a computer screen (the mirror #4 in the picture is just for you to see what they have seen on the computer monitor, which you cannot see in the video). If a word is presented on a red background (as in this example), then they have to write down the word on a sheet of paper on the desk (#5). If the word is not on a red background, they can ignore it.

- (6) They are also asked questions, which they have to respond to. They have been asked to give proper answers, as if they were chatting to someone, and to not give only short one-word answers.

Participants have been told that the Mixing task is the most important one. In the end, when the time is up, they will be assessed by how far they got in the Mixing task.

Every time the timer beeps, they have to work on tasks #2 and #3 before they can return to the Mixing task #1. This creates time pressure.*

Next, they were instructed on the seven questions they had to answer:

*Your task is to watch the people in the videos how they do the task. We then will ask you how they appeared to you. In other words, we are interested in the impression they left on you. We will ask you the following questions after each video:

- Their stress level: Does the person appear rather****Calm****or rather****Stressed****to you?

- How they managed to do the task: Do you think they****struggled****with the task, which means that they found it difficult to do and keep up. Or do you think they were****well in control****of the task, easily handling and managing it?

- Their enjoyment: Do you think they****disliked****the task, which means they did not enjoy doing it. Or do you think they****liked****(=enjoyed) doing the task?

- How they performed: You will not know whether they made mistakes or not in most tasks (but you will see whether they missed a word on red background, or missed to answer questions, or gave very short answers, even if you can't understand what they are saying). But we are interested in what you think how they performed, if you would have to guess. Did they****perform poorly****in the task, which means they made mistakes and did not make much progress in the Mixing task. Or do you think they****performed well****in the task, which means they made no or only few mistakes and made good progress in the Mixing task?

- Their motivation: Do you have the impression that they put****no effort at all****into doing the task well? Or do you have the impression they put****a lot of effort****into the task?

- About their physiological 'arousal': Do you have the impression that the person in the video is rather****sleepy and tired****, or are they rather****wide-awake and alert****?

- Their happiness: Do you think they are rather****Sad****, which means the are feeling negative. Or do you think they are rather****Happy****, which means they are feeling positive.****Important:****Don't think about the questions for too long. You do not need to explain them. Instead, just use your gut feeling and answer quickly. We are interested in your impression, which means how you feel about it.****On average, you should not take more than 20-30 seconds to answer all questions of one video.***

### Practice

After having been instructed as described above, they could practice the task with two videos. These videos were not part of the set of videos shown to the participants in the respective main study (in their run of the experiment), but taken from another of the eight runs (remember that the 160 videos were split into eight experimental runs, each run presenting 20 videos, resulting in a study duration of ~50 min per experimental run). The videos were selected based on two criteria: First, both videos either showed males or females. Four of the eight runs used males, four used females. Second, videos were chosen so that one video showed a person appearing (as judged by the author AJS) rather calm and low-stressed (usually from the beginning of the trial with low time pressure (LTP), always presented as first practice video), and the other video showed a person appearing rather stressed (usually from the end of the trial with high time pressure (LTP), always shown as second practice trial). This aimed to give participants an idea about the range of potential behaviours they might see in the main experiment.

### Balancing

One experimental run consisted of 20 videos. These 20 videos came from 10 different multitaskers (always five males and five females, except for run 8 which had videos of 4 males and 6 females). For each multitasker, their HTP and LTP clips were presented in the same experimental run.

Due to programming restrictions, the order of videos could not be randomized individually for each participant and, therefore, was the same within each experiment. The conditions were presented in a balanced way, according to the following criteria: Gender was always changed every two videos, for example male-male-female-female-male-male and so forth, and half of the experiments started with male videos, the other half with female videos. Within a set of two, there was always a high time pressure (HTP) and a low time pressure (LTP) video (example order: male-HTP, male-LTP, female-HTP, female-LTP, male-LTP, male-HTP, female-LTP, female-HTP, and then again male-HTP, and so forth). Half of the experiments started with HTP videos, the other half with LTP videos. Finally, the order of HTP and LTP was varied, so that for half of the persons shown in the videos (‘multitaskers’) first their LTP video was shown, and for the other half of the persons first their HTP video was shown. The same person was never shown in two videos in direct succession, and on average there were at least 5 other videos (~ 10 min) shown between the two videos of the same person.

# Additional Results Study 2

Based on the findings in Study 1, we predicted that the observer judgements would reflect that women are better at multitasking than men, e.g. that women appear more in control of the task and appear less stressed. Because not answering questions in the Conversation Task is easy to recognise for observers, we also predicted that the observer judgements would at least partially correlate with the Conversation Task performance.

## Full Statistics and graphs for the observer-rating analyses

In the following, comprehensive graphs and statistical analyses for the observer ratings are presented, separate for each question. First, 2x2-factorial repeated-measure ANOVAs (factors Sex and Time Pressure) are reported. These are then followed up with four paired-sample t-tests comparing all combinations in more detail to fully understand result patterns.

Panel (A) “Full Analysis” are the analyses reported in the main paper, but more comprehensive. These analyses are based on the full set of observers (N = 160) and all available videos (N = 160).

Panel (B) “Perfect Conversation Task” refer to the analysis reported in the main paper which excluded all videos in which a ‘multitasker’ (of Study 1) did not answer one or more questions. For this, we had to exclude 30% of the videos showing female multitaskers and 58% of the videos showing male multitaskers, leaving 53 female and 33 male videos (total N = 86). Due to these exclusions, in two runs of the experiment, no video of males performing under high time pressure (HTP) were left (all were excluded because they made mistakes), so that for this condition (males, HTP) no data were available. Because the ANOVA excludes these cases listwise, we also removed them from all ANOVA analyses and for the creation of the graphs. In other words, two experimental runs (N = 40 observers) were excluded, leaving N = 120 observers. To maximise statistical power, however, we kept them (and excluded these cases pairwise) for the t-tests, which explains that the t-tests have either 119 (if male-HTP was part of the comparison) or 159 degrees of freedom (if male-HTP was not part of the comparison).

Panel (C) “No Stereotype” refers to the following analysis: It is conceivable that some of the observers themselves believed in stereotypes about sex differences in multitasking abilities, and that these stereotypes affected their ratings. To rule this out, we conducted above 2x2 mixed ANOVAs with the factors Sex and Time Pressure again, but only using observers who self-reported to believe that neither males nor females are better at multitasking. In more detail, we included those observers who answered the question “What do you think, are men or women better at multitasking? (select "50" if you think both are the same)” with a score between 49 and 51 (scale 0 – 100) (note that this question was presented after observers rated the videos, i.e. towards the end of the experiment. Therefore, this question cannot have affected the ratings). This left 52 of the original 160 participants.

### Question 1 (Calm vs Stressed)

**(A) Full Analysis (B) Perfect Conversation Task (C) No Stereotype**


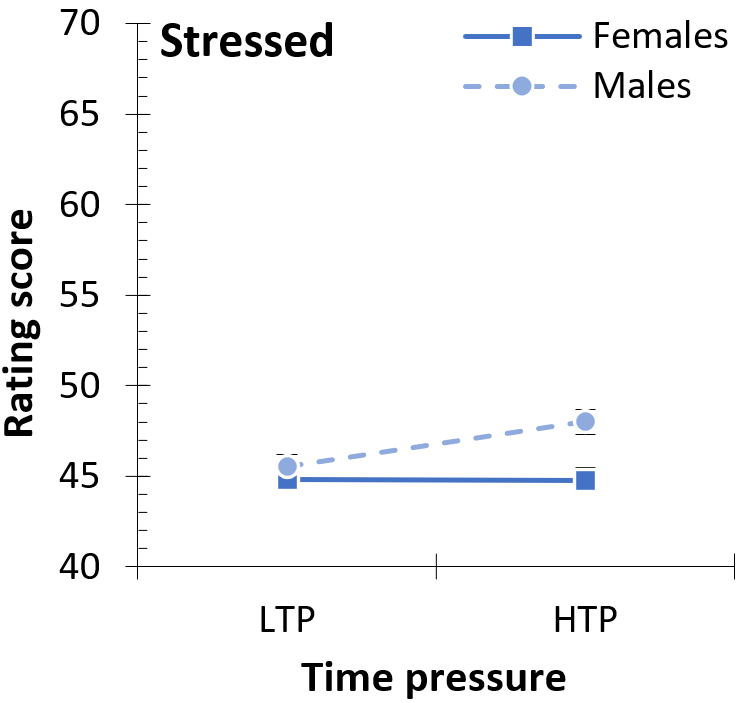

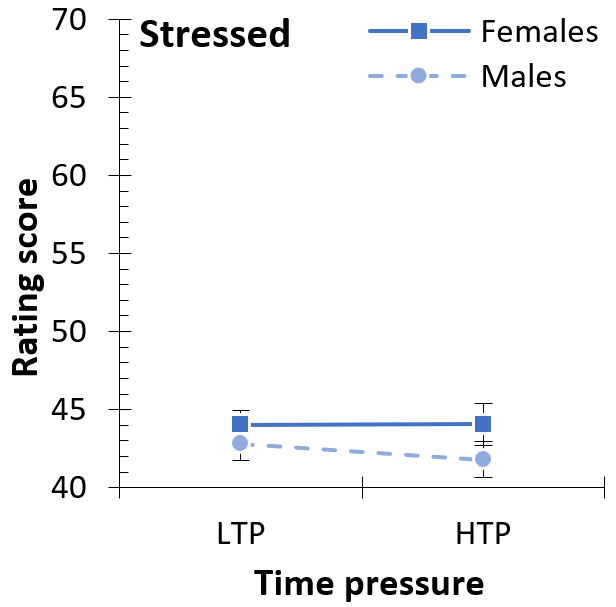

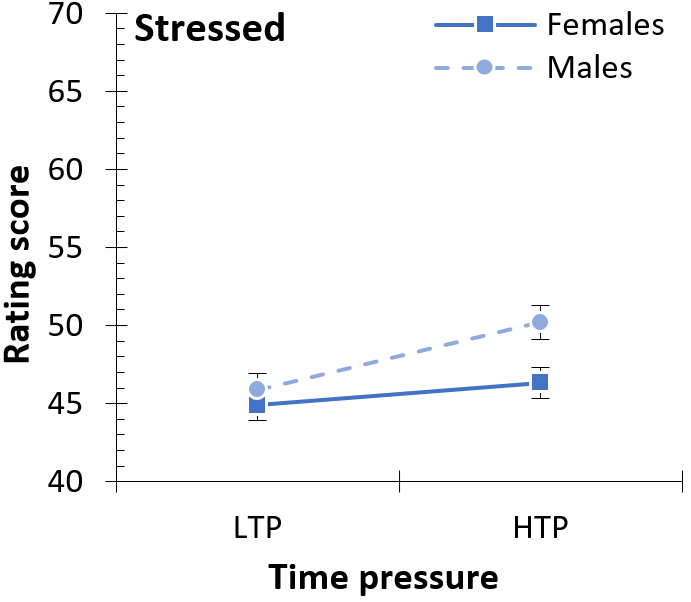


**Figure S1.** Detailed performance for Question 1 (calm/stressed).

**Table S2.** Descriptive statistics (Means, Standard Deviations)

| *Descriptive Statistics* | **(A) Full Analysis**  **(N=160; 160 videos)** | |  | **(B) Perfect Conv Task**  **(N=120^(*)^; 86 videos)** | |  | **(C) No Stereotypes**  **(N=52^(**)^; 160 videos)** | |
| --- | --- | --- | --- | --- | --- | --- | --- | --- |
|  | **LTP** | **HTP** |  | **LTP** | **HTP** |  | **LTP** | **HTP** |
| **Females** | 44.81  (SD 13.48) | 44.79  (SD 13.17) |  | 44.04  (SD 13.91) | 44.07  (SD 17.20) |  | 44.90  (SD 12.54) | 46.34  (SD 10.71) |
| **Males** | 45.53  (SD 12.48) | 48.01  (SD 12.71) |  | 42.83  (SD 17.15) | 41.81  (SD 16.97) |  | 45.90  (SD 11.78) | 50.20  (SD 10.53) |

*(*) There were fewer observers (N = 120 instead of N = 160) because for two experimental runs (each with 20 observers as participants), there were no “male-multitasker under high time pressure” videos left, i.e. in two experiments we had missing data.*

*(**) There was at least one participant in each of the eight experimental runs left, so that there were ratings for all videos.*

**Table S3.** Results of the 2x2 factorial repeated-measures ANOVAs with the factors Sex and Time Pressure. (ME = Main Effect)

| *ANOVA* | **(A) Full Analysis** | **(B) Perfect Conv Task** | **(C) No Stereotypes** |
| --- | --- | --- | --- |
| **ME Sex** | F(1,159)=3.675, p=.057, pη^2^=.023 | F(1,119)=1.286, p=.259, pη^2^=.011 | F(1,51)=3.034, p=.088, pη^2^=.056 |
| **ME Time Pressure** | F(1,159)=3.774, p=.054, pη^2^=.023 | F(1,119)=.155, p=.694, pη^2^=.001 | F(1,51)=5.665, **p=.021**, pη^2^=.100 |
| **Interaction** | F(1,159)=3.939, **p=.049**, pη^2^=.024 | F(1,119)=.208, p=.649, pη^2^=.002 | F(1,51)=2.771, p=.102, pη^2^=.052 |

**Table S4.** Results of paired-sample t-tests. Comparison of low time pressure (LTP) vs high time pressure (LTP) for females only (first row) and males only (second row). Comparison of females vs males, for LTP only (third row) and HTP only (fourth row).

| *t-tests* | **(A) Full Analysis** | **(B) Perfect Conv Task^(*)^** | **(C) No Stereotypes** |
| --- | --- | --- | --- |
| F: LTP vs HTP | t(159)=.014, p=.988, d=.001 | t(159)=.509, p=.611, d=.040 | t(51)=1.046, p=.300, d=.145 |
| M: LTP vs HTP | t(159)=2.775, **p=.006**, d=.219 | t(119)=.608, p=.544, d=.056 | t(51)=2.716, **p=.009**, d=.377 |
| LTP: F vs M | t(159)=.613, p=.541, d=.048 | t(159)=1.440, p=.152, d=.114 | t(51)=.612, p=.543, d=.085 |
| HTP: F vs M | t(159)=2.606, **p=.010**, d=.049 | t(119)=1.032, p=.304, d=.094 | t(51)=2.341, **p=.023**, d=.325 |

*(*) Degrees of freedom vary because of missing data for two experimental runs (40 participants in total) who did not have any data for male videos under high time pressure*

### Question 2 (In Control):

**(A) Full Analysis (B) Perfect Conversation Task (C) No Stereotype**


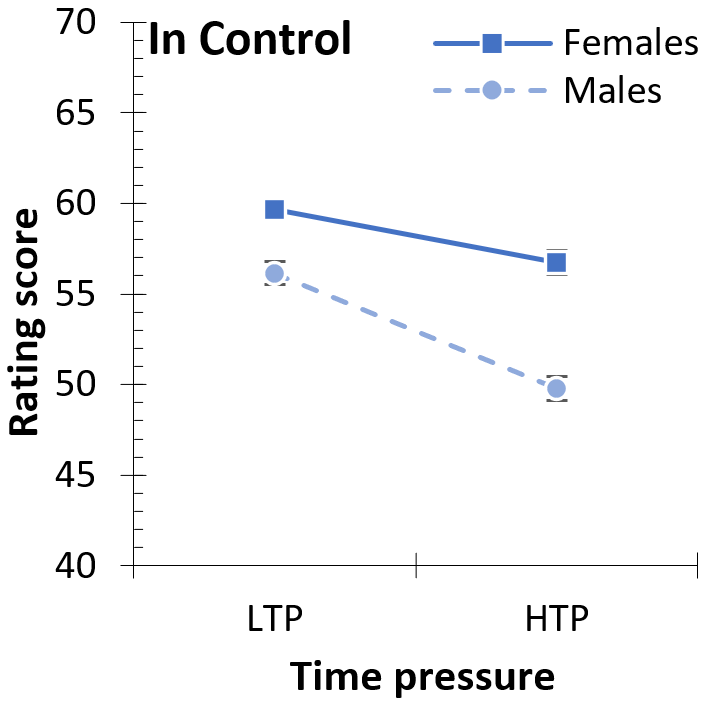

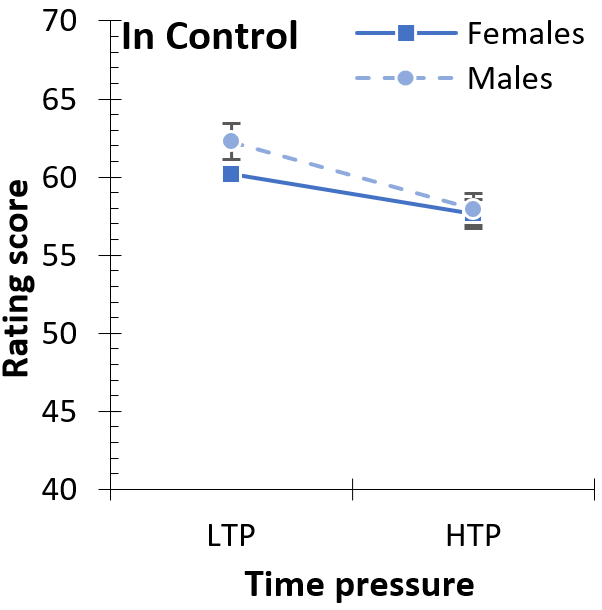

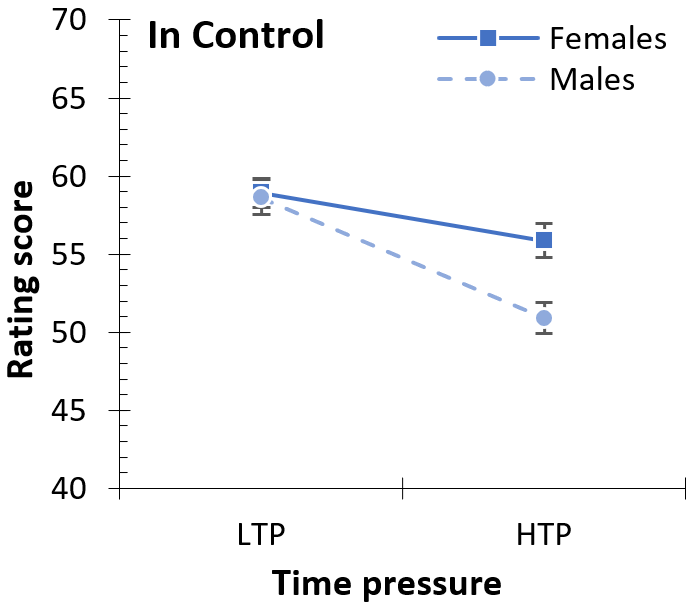


**Figure S2.** Detailed performance for Question 2 (In Control).

**Table S5.** Descriptive statistics (Means, Standard Deviations)

| *Descriptive Statistics* | **(A) Full Analysis**  **(N=160; 160 videos)** | |  | **(B) Perfect Conv Task**  **(N=120; 86 videos)** | |  | **(C) No Stereotypes**  **(N=52; 160 videos)** | |
| --- | --- | --- | --- | --- | --- | --- | --- | --- |
|  | **LTP** | **HTP** |  | **LTP** | **HTP** |  | **LTP** | **HTP** |
| **Females** | 59.67  (SD 12.83) | 56.75  (SD 12.47) |  | 60.17  (SD 13.27) | 57.65  (SD 17.09) |  | 58.94  (SD 12.83) | 55.87  (SD 12.08) |
| **Males** | 56.14  (SD 11.93) | 49.78  (SD 13.07) |  | 62.27  (SD 15.75) | 57.95  (SD 16.40) |  | 58.63  (SD 12.38) | 50.91  (SD 12.84) |

**Table S6.** Results of the 2x2 factorial repeated-measures ANOVAs with the factors Sex and Time Pressure.

| *ANOVA* | **(A) Full Analysis** | **(B) Perfect Conv Task** | **(C) No Stereotypes** |
| --- | --- | --- | --- |
| **ME Sex** | F(1,159)=31.159, **p<.001**, pη^2^=.164 | F(1,119)=.861, p=.355, pη^2^=.007 | F(1,51)=3.539, p=.066, pη^2^=.065 |
| **ME Time Pressure** | F(1,159)=48.647, **p<.001**, pη^2^=.234 | F(1,119)=9.873, **p=.002**, pη^2^=.077 | F(1,51)=20.102, **p<.001**, pη^2^=.283 |
| **Interaction** | F(1,159)=9.928, **p=.002**, pη^2^=.059 | F(1,119)=.535, p=.466, pη^2^=.004 | F(1,51)=7.446, **p=.009**, pη^2^=.127 |

**Table S7.** Results of paired-sample t-tests (see Table S4 for more details).

| *t-tests* | **(A) Full Analysis** | **(B) Perfect Conv Task** | **(C) No Stereotypes** |
| --- | --- | --- | --- |
| F: LTP vs HTP | t(159)=3.408, **p<.001**, d=.269 | t(159)=.157, p=.875, d=.012 | t(51)=2.122, **p=.039**, d=.294 |
| M: LTP vs HTP | t(159)=7.363, **p<.001**, d=.582 | t(119)=2.630, **p=.010**, d=.240 | t(51)=5.132, **p<.001**, d=.712 |
| LTP: F vs M | t(159)=3.347, **p=.001**, d=.265 | t(159)=1.353, p=.178, d=.107 | t(51)=.196, p=.846, d=.027 |
| HTP: F vs M | t(159)=6.233, **p<.001**, d=.493 | t(119)=.155, p=.877, d=.014 | t(51)=2.910, **p=.005**, d=.404 |

### Question 3 (Likes task):

**(A) Full Analysis (B) Perfect Conversation Task (C) No Stereotype**


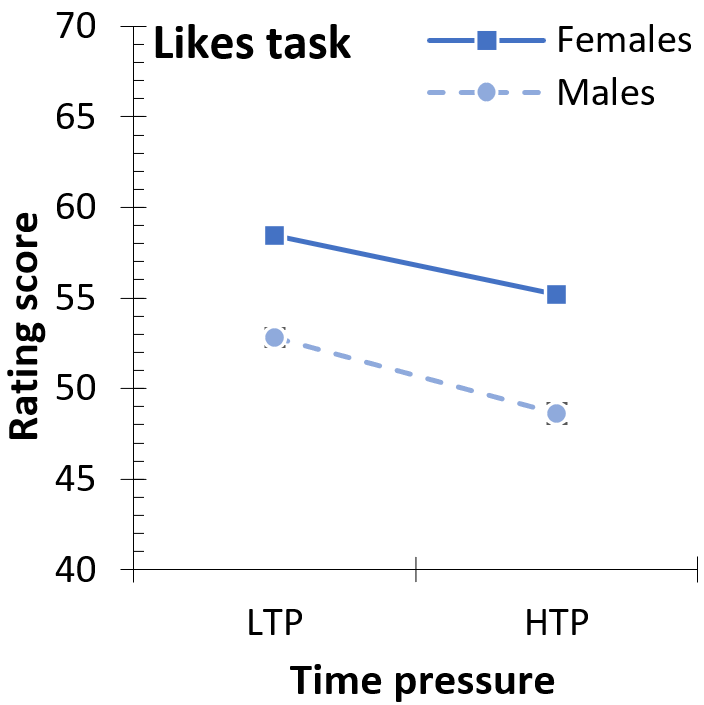

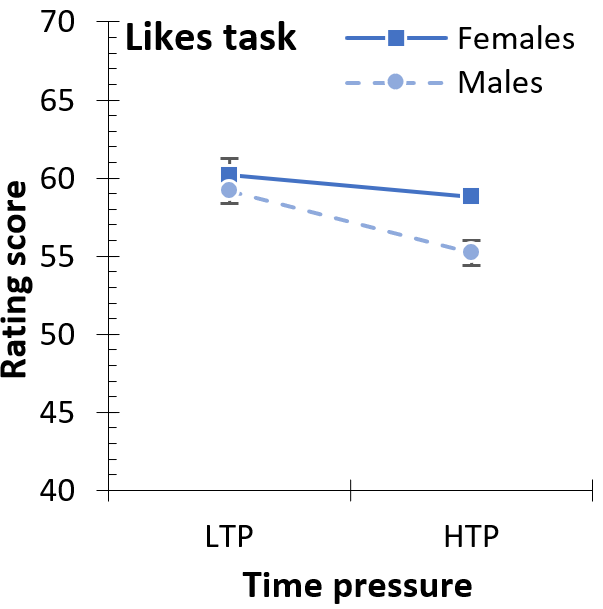

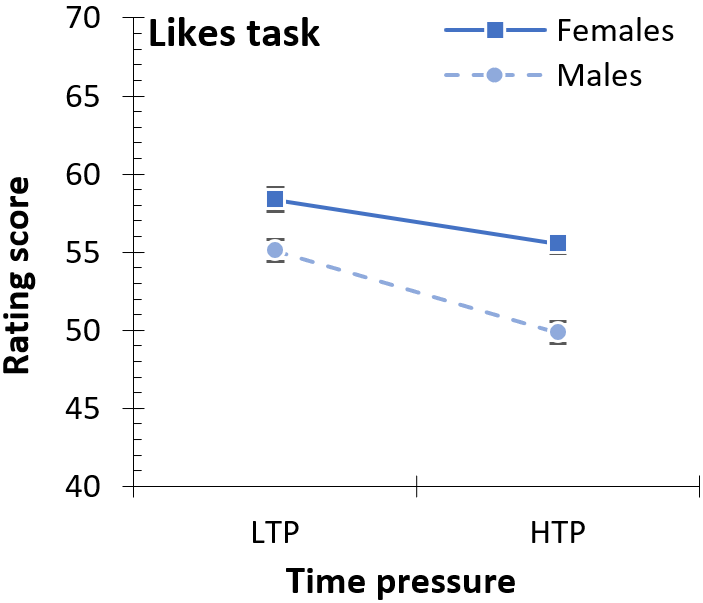


**Figure S3.** Detailed performance for Question 3 (Likes Task).

**Table S8.** Descriptive statistics (Means, Standard Deviations)

| *Descriptive Statistics* | **(A) Full Analysis**  **(N=160; 160 videos)** | |  | **(B) Perfect Conv Task**  **(N=120; 86 videos)** | |  | **(C) No Stereotypes**  **(N=52; 160 videos)** | |
| --- | --- | --- | --- | --- | --- | --- | --- | --- |
|  | **LTP** | **HTP** |  | **LTP** | **HTP** |  | **LTP** | **HTP** |
| **Females** | 58.48  (SD 13.67) | 55.18  (SD 11.94) |  | 60.18  (SD 14.55) | 58.80  (SD 15.37) |  | 58.37  (SD 13.79) | 55.54  (SD 12.07) |
| **Males** | 52.83  (SD 12.74) | 48.61  (SD 12.78) |  | 59.18  (SD 15.75) | 55.22  (SD 15.33) |  | 55.09  (SD 13.15) | 49.84  (SD 12.64) |

**Table S9.** Results of the 2x2 factorial repeated-measures ANOVAs with the factors Sex and Time Pressure.

| *ANOVA* | **(A) Full Analysis** | **(B) Perfect Conv Task** | **(C) No Stereotypes** |
| --- | --- | --- | --- |
| **ME Sex** | F(1,159)=57.624, **p<.001**, pη^2^=.266 | F(1,119)=4.689, **p=.032**, pη^2^=.038 | F(1,51)=20.665, **p<.001**, pη^2^=.288 |
| **ME Time Pressure** | F(1,159)=52.760, **p<.001**, pη^2^=.249 | F(1,119)=7.629, **p=.007**, pη^2^=.199 | F(1,51)=29.985, **p<.001**, pη^2^=.370 |
| **Interaction** | F(1,159)=.976, p=.325, pη^2^=.006 | F(1,119)=1.670, p=.199, pη^2^=.014 | F(1,51)=3.325, p=.074, pη^2^=.061 |

**Table S10.** Results of paired-sample t-tests (see Table S4 for more details).

| *t-tests* | **(A) Full Analysis** | **(B) Perfect Conv Task** | **(C) No Stereotypes** |
| --- | --- | --- | --- |
| F: LTP vs HTP | t(159)=4.668, **p<.001**, d=.369 | t(159)=.063, p=.950., d=.005 | t(51)=2.900, **p=.005**, d=.402 |
| M: LTP vs HTP | t(159)=6.190, **p<.001**, d=.489 | t(119)=2.551, **p=.012**, d=.233 | t(51)=5.230, **p<.001**, d=.722 |
| LTP: F vs M | t(159)=6.211, **p<.001**, d=.491 | t(159)=.264, p=.792, d=.021 | t(51)=2.599, **p=.012**, d=.360 |
| HTP: F vs M | t(159)=6.938, **p<.001**, d=.548 | t(119)=.2383, **p=.019**, d=.218 | t(51)=5.120, **p<.001**, d=.710 |

### Question 4 (Performance):

**(A) Full Analysis (B) Perfect Conversation Task (C) No Stereotype**


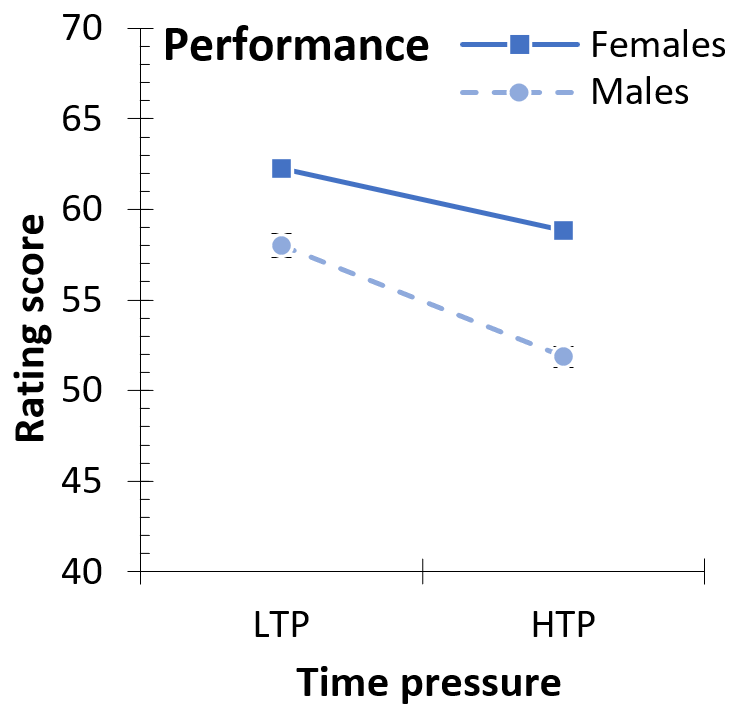

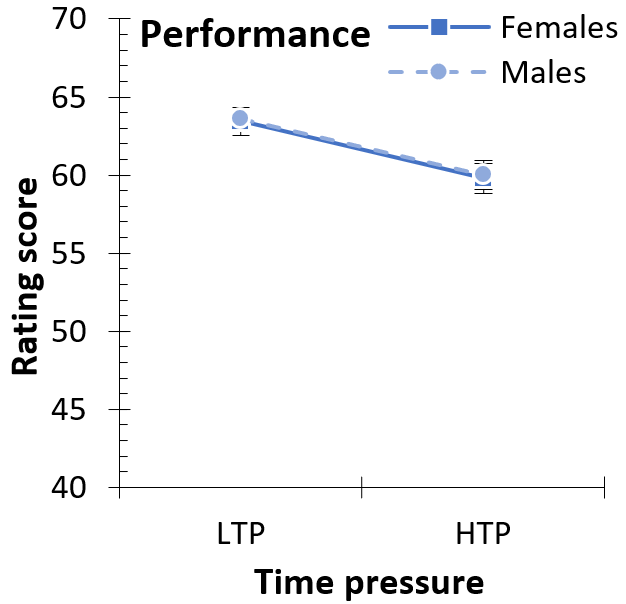

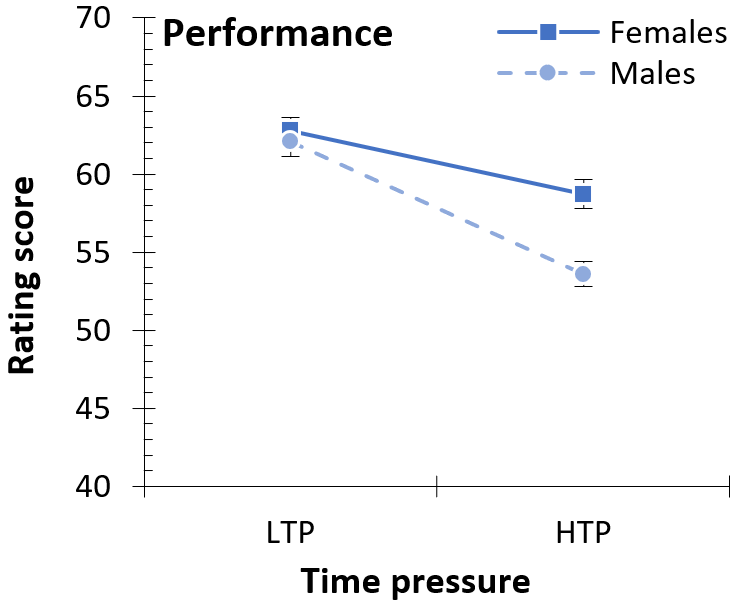


**Figure S4.** Detailed performance for Question 4 (Performance).

**Table S11.** Descriptive statistics (Means, Standard Deviations)

| *Descriptive Statistics* | **(A) Full Analysis**  **(N=160; 160 videos)** | |  | **(B) Perfect Conv Task**  **(N=120; 86 videos)** | |  | **(C) No Stereotypes**  **(N=52; 160 videos)** | |
| --- | --- | --- | --- | --- | --- | --- | --- | --- |
|  | **LTP** | **HTP** |  | **LTP** | **HTP** |  | **LTP** | **HTP** |
| **Females** | 62.26  (SD 12.98) | 58.86  (SD 12.81) |  | 63.44  (SD 13.53) | 59.79  (SD 15.85) |  | 62.76  (SD 12.64) | 58.72  (SD 11.45) |
| **Males** | 58.03  (SD 13.58) | 51.86  (SD 12.69) |  | 63.62  (SD 17.64) | 60.03  (SD 16.21) |  | 62.06  (SD 13.53) | 53.60  (SD 11.41) |

**Table S12.** Results of the 2x2 factorial repeated-measures ANOVAs with the factors Sex and Time Pressure.

| *ANOVA* | **(A) Full Analysis** | **(B) Perfect Conv Task** | **(C) No Stereotypes** |
| --- | --- | --- | --- |
| **ME Sex** | F(1,159)=38.002, **p<.001**, pη^2^=.193 | F(1,119)=.030, p=.862, pη^2^=.000 | F(1,51)=6.722, **p=.012**, pη^2^=.116 |
| **ME Time Pressure** | F(1,159)=48.466, **p<.001**, pη^2^=.234 | F(1,119)=12.495, **p<.001**, pη^2^=.095 | F(1,51)=34.529, **p<.001**, pη^2^=.404 |
| **Interaction** | F(1,159)=6.181, **p=.014**, pη^2^=.037 | F(1,119)=.001, p=.979, pη^2^=.000 | F(1,51)=7.203, **p=.010**, pη^2^=.124 |

**Table S13.** Results of paired-sample t-tests (see Table S4 for more details).

| *t-tests* | **(A) Full Analysis** | **(B) Perfect Conv Task** | **(C) No Stereotypes** |
| --- | --- | --- | --- |
| F: LTP vs HTP | t(159)=3.749, **p<.001**, d=.296 | t(159)=1.049, p=.296, d=.083 | t(51)=3.013, **p=.004**, d=.418 |
| M: LTP vs HTP | t(159)=7.145, **p<.001**, d=.565 | t(119)=2.260, **p=.026**, d=.206 | t(51)=6.278, **p<.001**, d=.871 |
| LTP: F vs M | t(159)=3.903, **p<.001**, d=.309 | t(159)=.164, p=.870, d=.013 | t(51)=.497, p=.311, d=.069 |
| HTP: F vs M | t(159)=6.652, **p<.001**, d=.526 | t(119)=.141, p=.888, d=.013 | t(51)=3.736, **p<.001**, d=.518 |

### Question 5 (Effort):

**(A) Full Analysis (B) Perfect Conversation Task (C) No Stereotype**


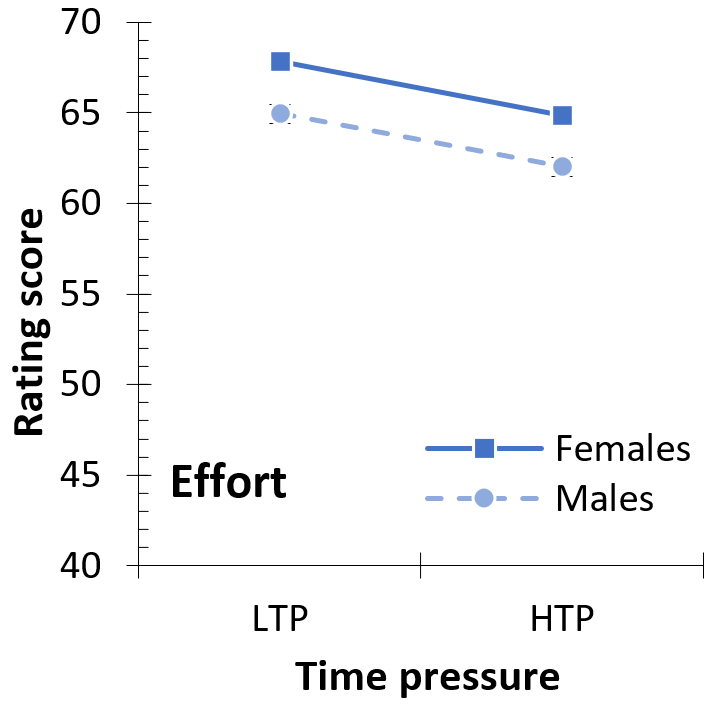

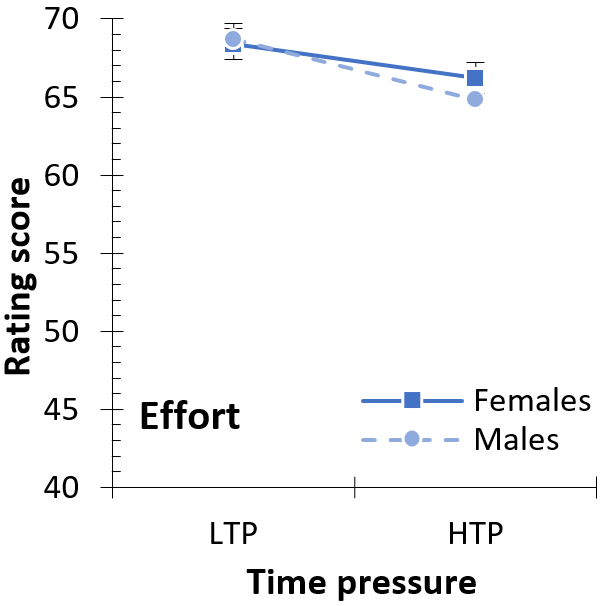

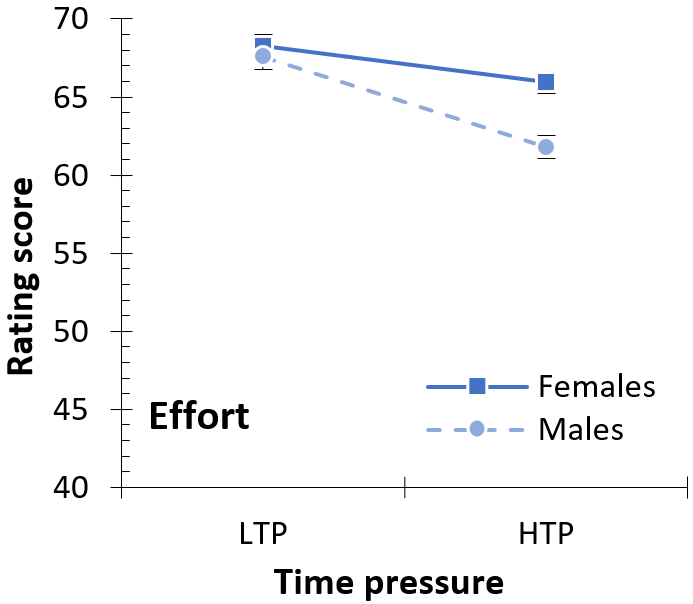


**Figure S5.** Detailed performance for Question 5 (Effort).

**Table S14.** Descriptive statistics (Means, Standard Deviations)

| *Descriptive Statistics* | **(A) Full Analysis**  **(N=160; 160 videos)** | |  | **(B) Perfect Conv Task**  **(N=120; 86 videos)** | |  | **(C) No Stereotypes**  **(N=52; 160 videos)** | |
| --- | --- | --- | --- | --- | --- | --- | --- | --- |
|  | **LTP** | **HTP** |  | **LTP** | **HTP** |  | **LTP** | **HTP** |
| **Females** | 67.85  (SD 13.87) | 64.84  (SD 13.43) |  | 68.39  (SD 15.03) | 66.20  (SD 15.27) |  | 68.27  (SD 12.09) | 65.95  (SD 12.29) |
| **Males** | 64.96  (SD 13.15) | 62.01  (SD 12.09) |  | 68.72  (SD 15.71) | 64.82  (SD 15.92) |  | 67.59  (SD 12.48) | 61.79  (SD 10.04) |

**Table S15.** Results of the 2x2 factorial repeated-measures ANOVAs with the factors Sex and Time Pressure.

| *ANOVA* | **(A) Full Analysis** | **(B) Perfect Conv Task** | **(C) No Stereotypes** |
| --- | --- | --- | --- |
| **ME Sex** | F(1,159)=15.458, **p<.001**, pη^2^=.089 | F(1,119)=.401, p=.528, pη^2^=.003 | F(1,51)=10.086, **p=.003**, pη^2^=.165 |
| **ME Time Pressure** | F(1,159)=25.988, **p<.001**, pη^2^=.140 | F(1,119)=18.485, **p<.001**, pη^2^=.134 | F(1,51)=17.815, **p<.001**, pη^2^=.259 |
| **Interaction** | F(1,159)=.004, p=.952, pη^2^=.000 | F(1,119)=1.136, p=.289, pη^2^=.009 | F(1,51)=4.590, **p=.037**, pη^2^=.083 |

**Table S16.** Results of paired-sample t-tests (see Table S4 for more details).

| *t-tests* | **(A) Full Analysis** | **(B) Perfect Conv Task** | **(C) No Stereotypes** |
| --- | --- | --- | --- |
| F: LTP vs HTP | t(159)=3.931, **p<.001**, d=.311 | t(159)=1.306, p=.194, d=.103 | t(51)=1.987, p=.052, d=.276 |
| M: LTP vs HTP | t(159)=3.832, **p<.001**, d=.303 | t(119)=3.574, **p<.001**, d=.326 | t(51)=4.323, **p<.001**, d=.599 |
| LTP: F vs M | t(159)=3.346, **p=.001**, d=.265 | t(159)=.150, p=.881, d=.012 | t(51)=.577, p=.567, d=.193 |
| HTP: F vs M | t(159)=3.147, **p=.002**, d=.249 | t(119)=1.165, p=.246, d=.106 | t(51)=4.005, **p<.001**, d=.261 |

### Question 6 (Alertness):

**(A) Full Analysis (B) Perfect Conversation Task (C) No Stereotype**


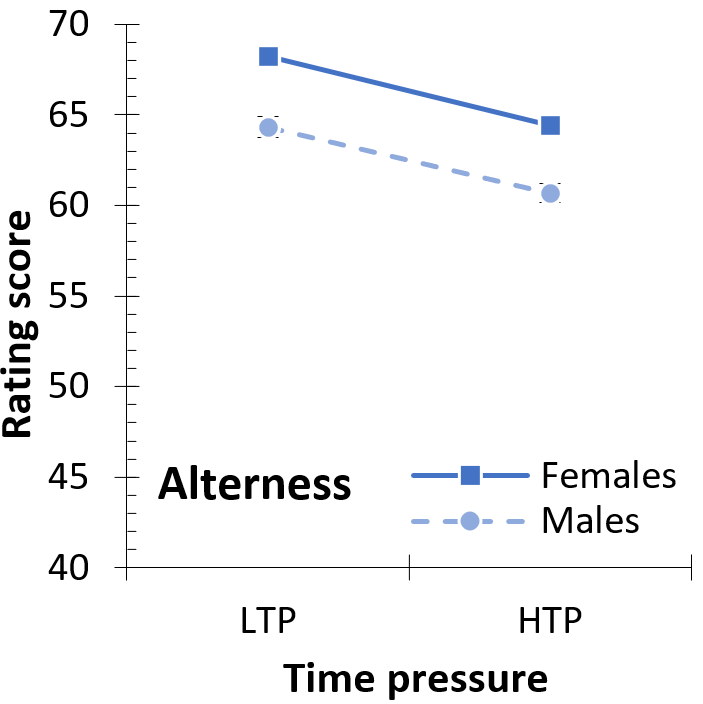

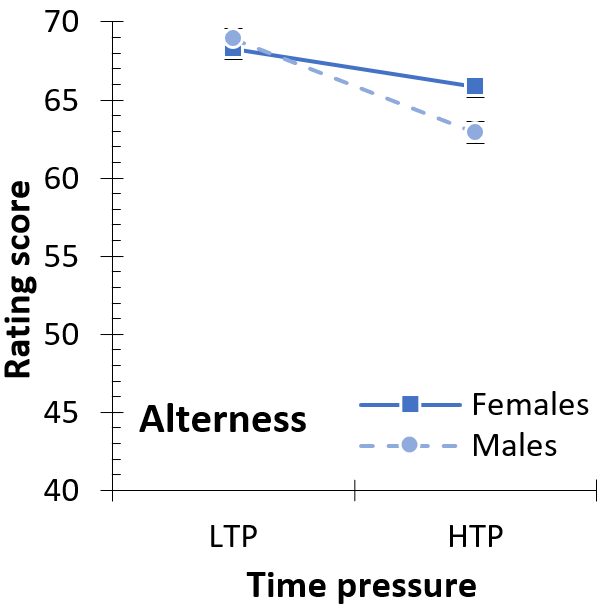

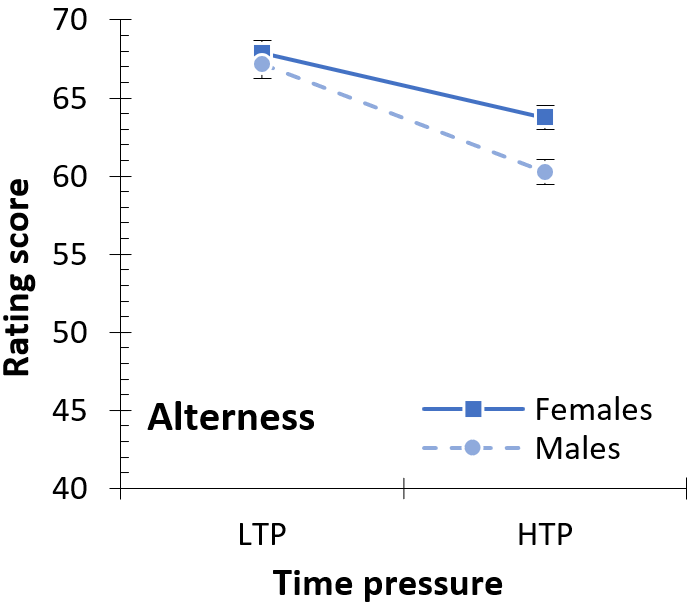


**Figure S6.** Detailed performance for Question 6 (Alertness).

**Table S17.** Descriptive statistics (Means, Standard Deviations)

| *Descriptive Statistics* | **(A) Full Analysis**  **(N=160; 160 videos)** | |  | **(B) Perfect Conv Task**  **(N=120; 86 videos)** | |  | **(C) No Stereotypes**  **(N=52; 160 videos)** | |
| --- | --- | --- | --- | --- | --- | --- | --- | --- |
|  | **LTP** | **HTP** |  | **LTP** | **HTP** |  | **LTP** | **HTP** |
| **Females** | 68.20  (SD 14.51) | 64.43  (SD 14.70) |  | 68.28  (SD 15.60) | 65.84  (SD 17.14) |  | 67.89  (SD 13.29) | 63.75  (SD 13.61) |
| **Males** | 64.32  (SD 15.52) | 60.67  (SD 14.74) |  | 68.93  (SD 17.53) | 62.92  (SD 17.94) |  | 67.16  (SD 14.55) | 60.27  (SD 12.62) |

**Table S18.** Results of the 2x2 factorial repeated-measures ANOVAs with the factors Sex and Time Pressure.

| *ANOVA* | **(A) Full Analysis** | **(B) Perfect Conv Task** | **(C) No Stereotypes** |
| --- | --- | --- | --- |
| **ME Sex** | F(1,159)=20.779, **p<.001**, pη^2^=.116 | F(1,119)=1.090, p=.299, pη^2^=.009 | F(1,51)=3.717, p=.059, pη^2^=.068 |
| **ME Time Pressure** | F(1,159)=42.301, **p<.001**, pη^2^=.210 | F(1,119)=18.944, **p<.001**, pη^2^=.137 | F(1,51)=41.680, **p<.001**, pη^2^=.450 |
| **Interaction** | F(1,159)=.020, p=.887, pη^2^=.000 | F(1,119)=3.812, p=.053, pη^2^=.031 | F(1,51)=2.272, p=.138, pη^2^=.043 |

**Table S19.** Results of paired-sample t-tests (see Table S4 for more details).

| *t-tests* | **(A) Full Analysis** | **(B) Perfect Conv Task** | **(C) No Stereotypes** |
| --- | --- | --- | --- |
| F: LTP vs HTP | t(159)=5.219, **p<.001**, d=.413 | t(159)=1.019, p=.310, d=.081 | t(51)=3.512, **p<.001**, d=.487 |
| M: LTP vs HTP | t(159)=5.042, **p<.001**, d=.399 | t(119)=4.313, **p<.001**, d=.394 | t(51)=5.208, **p<.001**, d=.722 |
| LTP: F vs M | t(159)=4.118, **p<.001**, d=.326 | t(159)=.457, p=.324, d=.036 | t(51)=.482, p=.632, d=.067 |
| HTP: F vs M | t(159)=3.936, **p<.001**, d=.311 | t(119)=1.843, **p=.034**, d=.168 | t(51)=2.596, **p=.012**, d=.360 |

### Question 7 (Happiness):

**(A) Full Analysis (B) Perfect Conversation Task (C) No Stereotype**


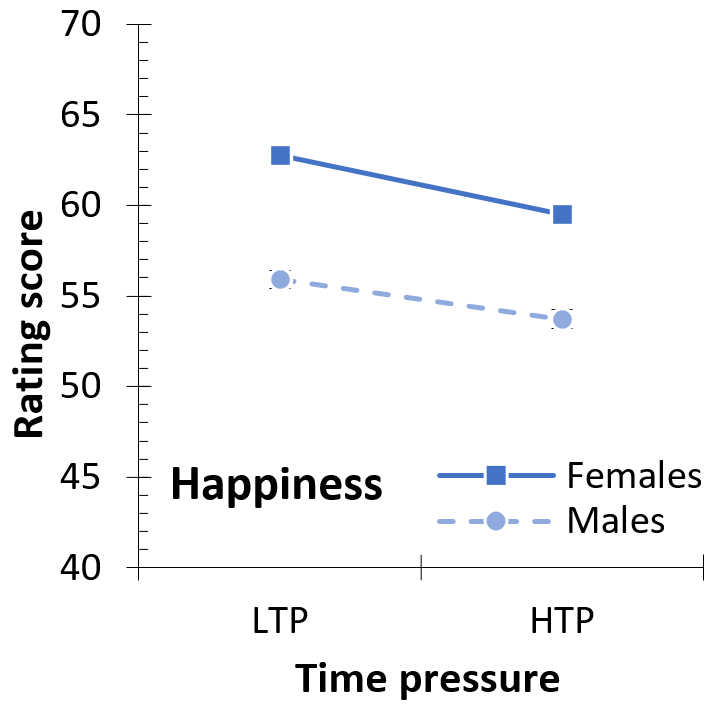

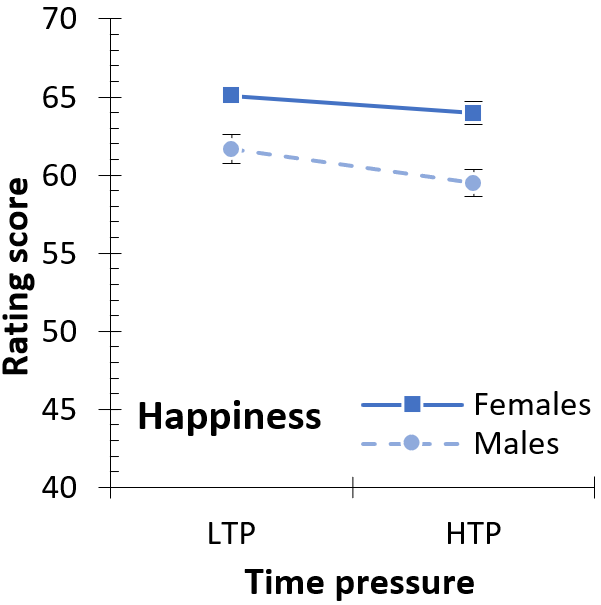

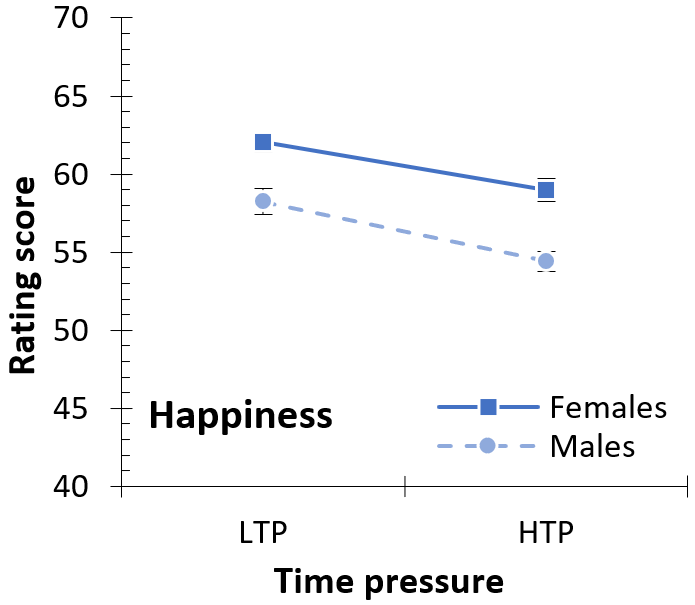


**Figure S7.** Detailed performance for Question 7 (Happiness).

**Table S20.** Descriptive statistics (Means, Standard Deviations)

| *Descriptive Statistics* | **(A) Full Analysis**  **(N=160; 160 videos)** | |  | **(B) Perfect Conv Task**  **(N=120; 86 videos)** | |  | **(C) No Stereotypes**  **(N=52; 160 videos)** | |
| --- | --- | --- | --- | --- | --- | --- | --- | --- |
|  | **LTP** | **HTP** |  | **LTP** | **HTP** |  | **LTP** | **HTP** |
| **Females** | 62.73  (SD 11.23) | 59.51  (SD 10.55) |  | 65.08  (SD 12.87) | 63.97 (SD 13.85) |  | 62.04  (SD 10.71) | 58.98  (SD 09.15) |
| **Males** | 55.92  (SD 11.82) | 53.73  (SD 10.45) |  | 61.66  (SD 14.79) | 59.48  (SD 13.37) |  | 58.24  (SD 11.57) | 54.43  (SD 09.89) |

**Table S21.** Results of the 2x2 factorial repeated-measures ANOVAs with the factors Sex and Time Pressure.

| *ANOVA* | **(A) Full Analysis** | **(B) Perfect Conv Task** | **(C) No Stereotypes** |
| --- | --- | --- | --- |
| **ME Sex** | F(1,159)=62.250, **p<.001**, pη^2^=.281 | F(1,119)=11.068, **p=.001**, pη^2^=.085 | F(1,51)=15.576, **p<.001**, pη^2^=.234 |
| **ME Time Pressure** | F(1,159)=30.792, **p<.001**, pη^2^=.162 | F(1,119)=3.387, p=.068, pη^2^=.028 | F(1,51)=22.358, **p<.001**, pη^2^=.305 |
| **Interaction** | F(1,159)=1.595, p=.209, pη^2^=.010 | F(1,119)=.435, p=.511, pη^2^=.004 | F(1,51)=.372, p=.545, pη^2^=.007 |

**Table S22.** Results of paired-sample t-tests (see Table S4 for more details).

| *t-tests* | **(A) Full Analysis** | **(B) Perfect Conv Task** | **(C) No Stereotypes** |
| --- | --- | --- | --- |
| F: LTP vs HTP | t(159)=4.918, **p<.001**, d=.389 | t(159)=.070, p=.944, d=.006 | t(51)=3.511, **p<.001**, d=.487 |
| M: LTP vs HTP | t(159)=3.558, **p<.001**, d=.281 | t(119)=1.664, p=.099, d=.152 | t(51)=3.725, **p<.001**, d=.517 |
| LTP: F vs M | t(159)=7.515, **p<.001**, d=.594 | t(159)=2.471, **p=.015**, d=.195 | t(51)=2.931, **p=.005**, d=.406 |
| HTP: F vs M | t(159)=6.524, **p<.001**, d=.516 | t(119)=3.105, **p=.002**, d=.283 | t(51)=3.984, **p<.001**, d=.553 |

## Correlations between Conversation Task performance and ratings

**Table S23.** Correlations of the performance in the five tasks in Study 1 with the observer ratings in Study 2. Across all participants, slightly different N are caused by missing performance data in Study 1. **Here, the Conversation Task was coded as z-transformed number of *missed answers*, i.e. higher z-scores reflect worse performance.** Cells shaded grey show statistically significant correlations.

|  | Conversation  N=68 ^(*)^ | Cooking  N=65 | Phone Numb.  N=68 | Numb-Letter  N=67 | Word Monit.  N=68 | Average^(**)^  N=64 |
| --- | --- | --- | --- | --- | --- | --- |
| Q1  Stress | r=.450  p<.001 | r=.037  p=.772 | r=-.120  p=.331 | r=-.002  p=.989 | r=.057  p=.645 | r=-.164  p=.796 |
| Q2  In Contr. | r=-.559  p<.001 | r=.142  p=.256 | r=.079  p=.520 | r=-.019  p=.613 | r=-.063  p=.613 | r=.222  p=.069 |
| Q3  Liking | r=-.561  p<.001 | r=-.059  p=.641 | r=.037  p=.762 | r=-.112  p=.366 | r=.005  p=.966 | r=.094  p=.446 |
| Q4  Perform | r=-.541  p<.001 | r=.068  p=.591 | r=.101  p=.410 | r=-.074  p=.554 | r=.036  p=.771 | r=.211  p=.084 |
| Q5  Effort | r=-.132  p=.284 | r=.066  p=.600 | r=-.034  p=.782 | r=-.160  p=.197 | r=.041  p=.744 | r=-.017  p=.891 |
| Q6  Alert | r=-.185  p=.130 | r=.024  p=.847 | r=-.071  p=.563 | r=-.156  p=.208 | r=.025  p=.844 | r=-.040  p=.747 |
| Q7  Happy | r=-.459  p<.001 | r=.010  p=.937 | r=-.025  p=.840 | r=-.123  p=.322 | r=-.002  p=.987 | r=.052  p=.673 |

(*) For the correlation between the Conversation Task performance and the questions, three datapoints were identified as outliers (z-scores of 3.59, 3.59 and 4.11) and removed from the analyses. However, results do not change if the outliers are not removed.

(**) Average performance across all five tasks (average z-score)

Note: The performance data in the tasks are taken from Study 1 and reflect performance across the whole 10-minute lasting trial. The ratings of the questions from Study 2 referred to two video clips of 75s each (LTP and HTP). For this correlational analysis, we first averaged the ratings for the LTP and HTP clip of each multitasker for one average score per multitasker.

**Figure S8.** Scatterplots for the correlations between the z-transformed Conversation Task performance (“ConvZ”) of the multitaskers in Study 1 and the ratings of the observers in Study 2. **Here, the Conversation Task was coded as z-transformed number of *missed answers*, i.e. higher z-scores reflect worse performance.** These plots illustrate the data in Table S23, column 1.

**(2) In Control**

*r* = -.559 ; p < .001; R^2^ = .312

**(1) Stressed**

*r* = .450; p < .001; R^2^ = .202


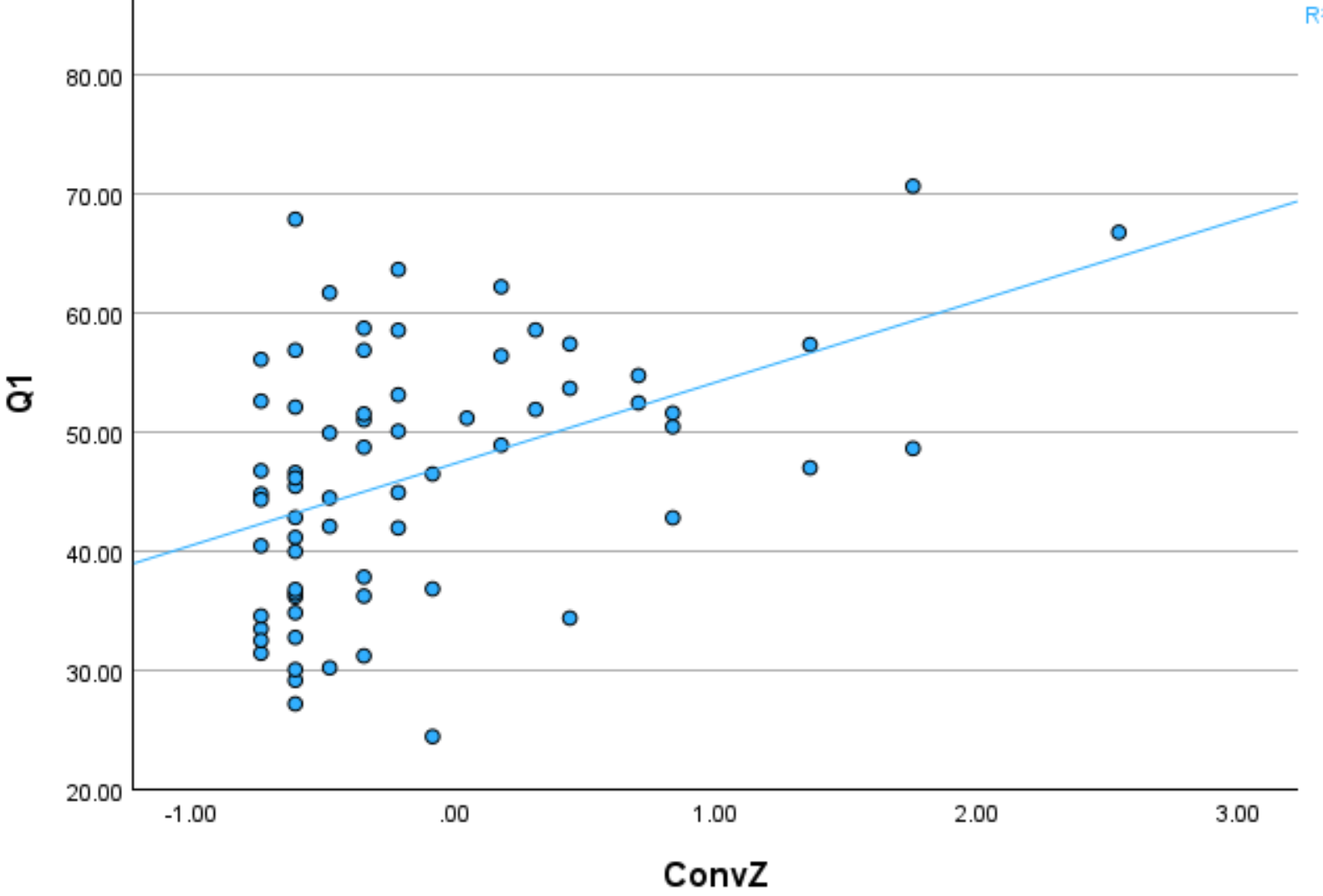

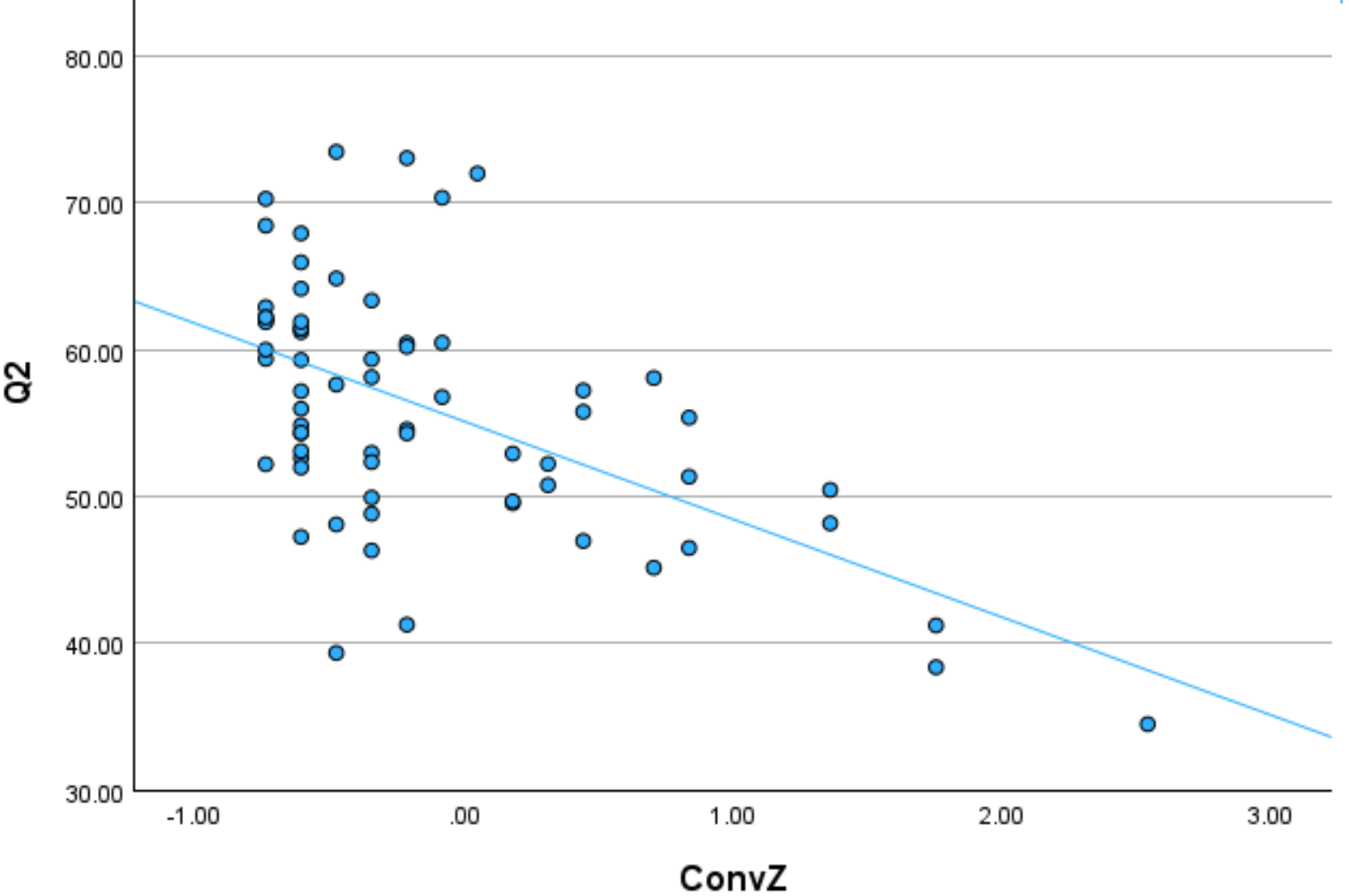


**(4) Performance**

*r* = -.541 ; p < .001; R^2^ = .292

**(3) Likes Task**

*r* = -.561 ; p < .001; R^2^ = .315


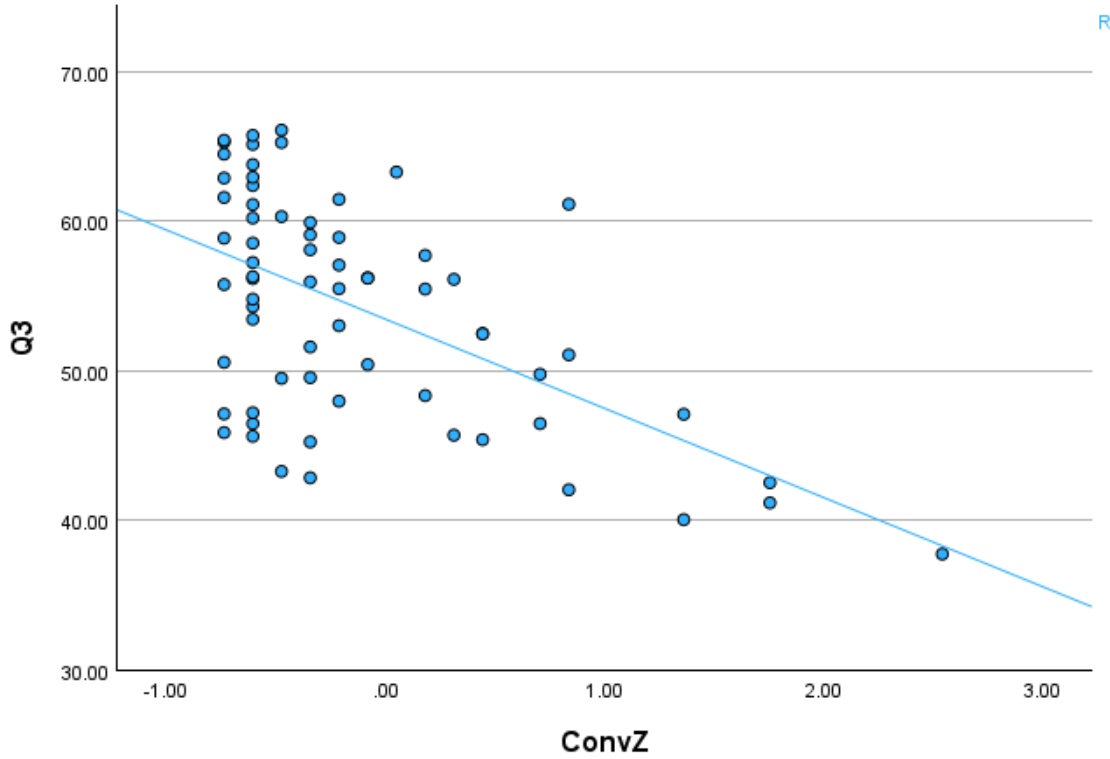

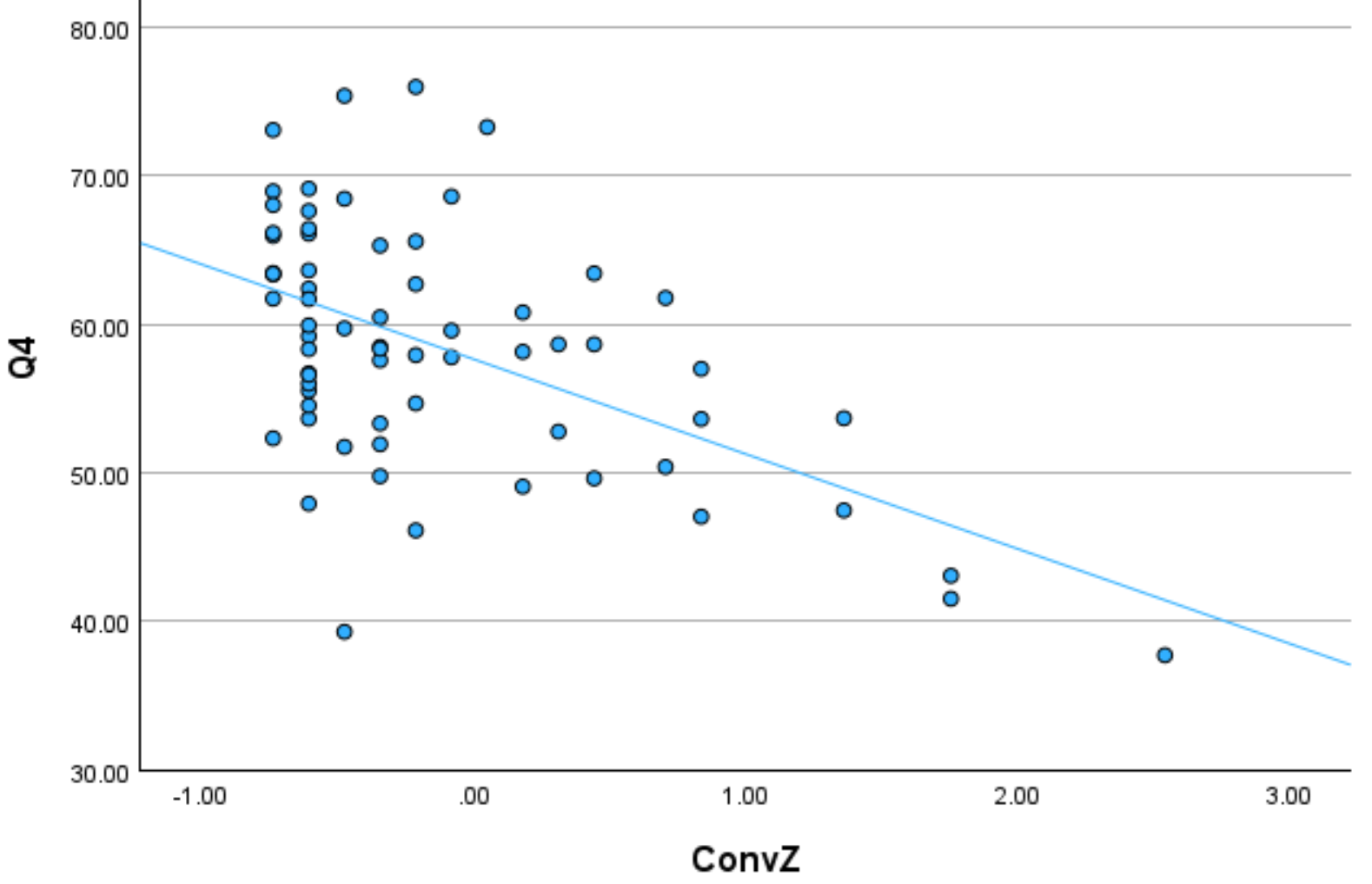


**(6) Alertness**

*r* = -.185, p=.130, R^2^ = .034

**(5) Effort**

*r* = -.132 ; p = .284; R^2^ = .017


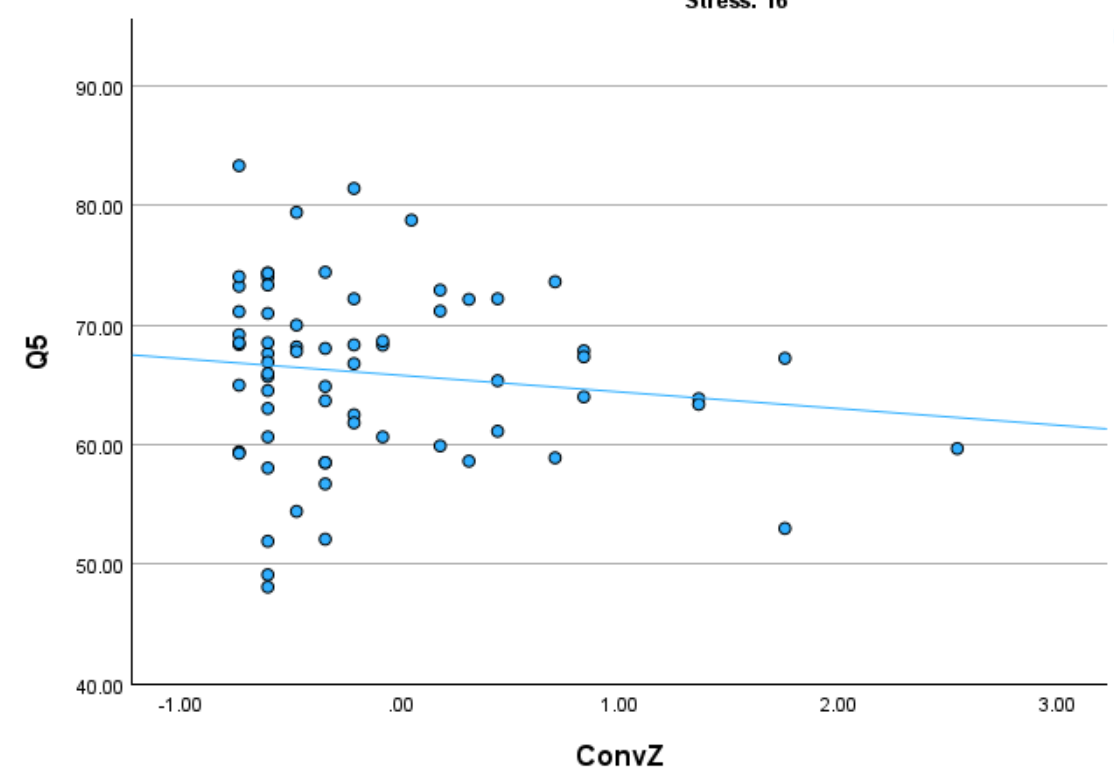

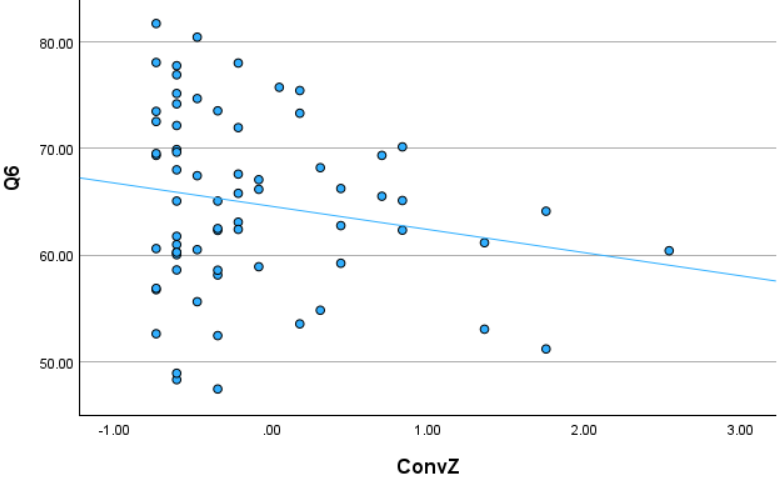


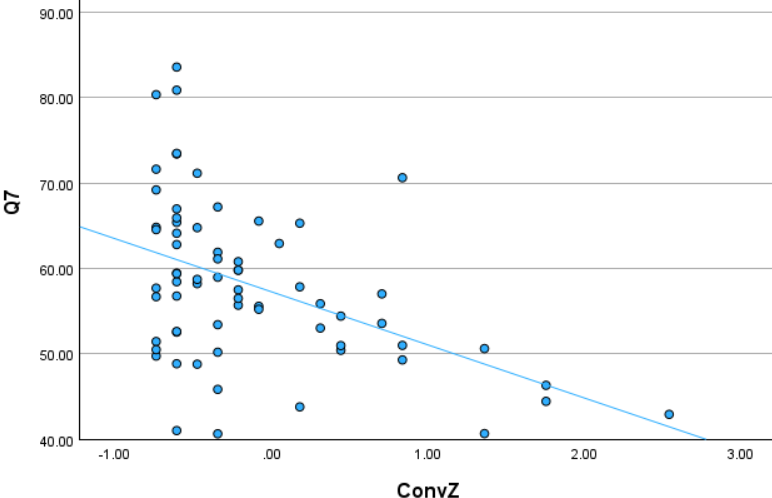


**(7) Happiness**

*r* = -.459, p<.001, R^2^ = .210

In Study 1, we explained that a naïve rater evaluated the two 75s video excerpts regarding the quality and the speed (promptness) of the answers to test whether there are sex differences not only in the number of missed answers, but potentially also in the quality and speed of the given answers. While there were no sex differences in the quality or speed of answers (see Study 1), across male and female participants those measures did correlate with most questions (Table S24; Quality of answers with Question 2 – 7, all r > .227, all p < .043; Question 1 r = -.190, p = .092; Speed of answers with Questions 2 – 5, all r > .276, all p < .013; Question 1, 6, 7 all r < .176, all p > .119). This shows that quality and speed of answers were indeed used by the observers to make their ratings, but that there were no sex differences.

**Table S24.** Shows correlations between the observer-ratings for Questions 1 – 7 and the Conversation Task performance (% of questions answered: how many questions had been answered in the two 75s clips (LTP and HTP), Quality/Speed: the quality and promptness of the answer as evaluated by a naïve rater. Cells shaded grey indicate significant correlations.

|  | % of questions answered, N=77 | Quality  N=80 | Speed  N=80 |
| --- | --- | --- | --- |
| Question 1  Stress | r=-.275  p=.016 | r=-.190  p=.092 | r=-.176  p=.119 |
| Question 2  In Control | r=.558  p<.001 | r=.389  p<.001 | r=.475  p<.001 |
| Question 3  Liking | r=.545  p<.001 | r=.510  p<.001 | r=.276  p=.013 |
| Question 4  Performance | r=.579  p<.001 | r=.436  p<.001 | r=.470  p<.001 |
| Question 5  Effort | r=.290  p=.011 | r=.295  p=.008 | r=.327  p=.003 |
| Question 6  Alertness | r=.233  p=.041 | r=.227  p=.043 | r=.154  p=.174 |
| Question 7  Happiness | r=.383  p<.001 | r=.384  p<.001 | r=.096  p=.396 |

**References**

Beede, K. E., & Kass, S. J. (2006). Engrossed in conversation: The impact of cell phones on simulated driving performance. *Accident Analysis & Prevention*, *38*(2), 415–421. https://doi.org/10.1016/j.aap.2005.10.015

Boboc, R. G., Voinea, G. D., Buzdugan, I.-D., & Antonya, C. (2022). Talking on the phone while driving: A literature review on driving simulator studies. *International Journal of Environmental Research and Public Health*, *19*(17), 10554.

Burns, P. C., Parkes, A., Burton, S., Smith, R. K., & Burch, D. (2002). *How Dangerous is Driving with a Mobile Phone?: Benchmarking the Impairment to Alcohol*. https://www.academia.edu/download/37690816/TRL547_-_How_dangerous_is_driving_with_a_mobile_phone__Benchmarking_the_impairment_to_alcohol_1_.pdf

Cao, S., & Liu, Y. (2013). Gender Factor in Lane Keeping and Speech Comprehension Dual Tasks. *Proceedings of the Human Factors and Ergonomics Society Annual Meeting*, *57*(1), 1909–1913. https://doi.org/10.1177/1541931213571426

Charlton, S. G. (2009). Driving while conversing: Cell phones that distract and passengers who react. *Accident Analysis & Prevention*, *41*(1), 160–173. https://doi.org/10.1016/j.aap.2008.10.006

Collet, C., Guillot, A., & Petit, C. (2010). Phoning while driving II: A review of driving conditions influence. *Ergonomics*, *53*(5), 602–616. https://doi.org/10.1080/00140131003769092

Drews, F. A., Pasupathi, M., & Strayer, D. L. (2008). Passenger and cell phone conversations in simulated driving. *Journal of Experimental Psychology: Applied*, *14*(4), 392.

Himi, S. A., Volberg, G., Bühner, M., & Hilbert, S. (2023). Individual differences in everyday multitasking behavior and its relation to cognition and personality. *Psychological Research*, *87*(3), 655–685. https://doi.org/10.1007/s00426-022-01700-z

Hirnstein, M., Larøi, F., & Laloyaux, J. (2019). No sex difference in an everyday multitasking paradigm. *Psychological Research*, *83*(2), 286–296. https://doi.org/10.1007/s00426-018-1045-0

Hirsch, P., Nolden, S., Philipp, A. M., & Koch, I. (2018). Hierarchical task organization in dual tasks: Evidence for higher level task representations. *Psychological Research*, *82*(4), 759–770. https://doi.org/10.1007/s00426-017-0851-0

Laguë-Beauvais, M., Gagnon, C., Castonguay, N., & Bherer, L. (2013). Individual differences effects on the psychological refractory period. *SpringerPlus*, *2*(1), 368. https://doi.org/10.1186/2193-1801-2-368

Logie, R. H., Trawley, S., & Law, A. (2011). Multitasking: Multiple, domain-specific cognitive functions in a virtual environment. *Memory & Cognition*, *39*(8), 1561–1574. https://doi.org/10.3758/s13421-011-0120-1

Lui, K. F., Yip, K. H., & Wong, A. C.-N. (2021). Gender differences in multitasking experience and performance. *Quarterly Journal of Experimental Psychology*, *74*(2), 344–362. https://doi.org/10.1177/1747021820960707

Mantyla, T. (2013). Gender differences in multitasking reflect spatial ability. *Psychological Science*, *24*(4), 514–520. https://doi.org/10.1177/0956797612459660

Redick, T. S., Shipstead, Z., Meier, M. E., Montroy, J. J., Hicks, K. L., Unsworth, N., Kane, M. J., Hambrick, D. Z., & Engle, R. W. (2016). Cognitive predictors of a common multitasking ability: Contributions from working memory, attention control, and fluid intelligence. *Journal of Experimental Psychology: General*, *145*(11), 1473–1492. https://doi.org/10.1037/xge0000219

Ren, D., Zhou, H., & Fu, X. (2009). A Deeper Look at Gender Difference in Multitasking: Gender-Specific Mechanism of Cognitive Control. *2009 Fifth International Conference on Natural Computation*, *5*, 13–17. https://doi.org/10.1109/ICNC.2009.542

Santiago-Espada, Y., Myer, R. R., Latorella, K. A., & Comstock, J. R. (n.d.). *The Multi-Attribute Task Battery II (MATB-II) Software for Human Performance and Workload Research: A User’s Guide*.

Stoet, G., O’Connor, D. B., Conner, M., & Laws, K. R. (2013). Are women better than men at multi-tasking? *BMC Psychology*, *1*(18), 1–10.

Szameitat, A. J., Hamaida, Y., Tulley, R. S., Saylik, R., & Otermans, P. C. (2015). ‘Women Are Better Than Men’-Public Beliefs on Gender Differences and Other Aspects in Multitasking. *PLoS One*, *10*(10), e0140371. https://doi.org/10.1371/journal.pone.0140371
